# YBX1 promotes type H vessel–dependent bone formation in an m5C-dependent manner

**DOI:** 10.1172/jci.insight.172345

**Published:** 2024-02-22

**Authors:** Yu-Jue Li, Qi Guo, Ming-Sheng Ye, GuangPing Cai, Wen-Feng Xiao, Sheng Deng, Ye Xiao

**Affiliations:** 1 Department of Endocrinology, Endocrinology Research Center;; 2 Department of Orthopedics; and; 3 Department of Pharmacy, Xiangya Hospital of Central South University, Changsha, China.

**Keywords:** Angiogenesis, Bone biology, Bone marrow differentiation, Osteoporosis

## Abstract

RNA-binding proteins (RBPs) interact with RNA and ubiquitously regulate RNA transcripts during their life cycle, playing a fundamental role in the progression of angiogenesis-related diseases. In the skeletal system, endothelium-dependent angiogenesis is indispensable for bone formation. However, the role of RBPs in endothelium-dependent bone formation is unclear. Here, we show that RBP–Y-box-binding protein 1 (YBX1) was strongly reduced in the bone vasculature of ovariectomy (OVX) mice. Endothelial cell–specific deletion of *Ybx1* impaired CD31-high, endomucin-high (CD31^hi^EMCN^hi^) endothelium morphology and resulted in low bone mass whereas *Ybx1* overexpression promoted angiogenesis-dependent osteogenesis and ameliorated bone loss. Mechanistically, *YBX1* deletion disrupted *CD31*, *EMCN*, and bone morphogenetic protein 4 (*BMP4*) stability in an m5C-dependent manner and blocked endothelium-derived BMP4 release, thereby inhibiting osteogenic differentiation of bone mesenchymal stromal cells. Administration of recombinant BMP4 protein restored impaired bone formation in *Ybx1* deletion mice. Tail vein injection of CD31-modified polyethylene glycol–poly (lactic-co-glycolic acid) carrying sciadopitysin, a natural YBX1 agonist, pharmacologically partially reversed CD31^hi^EMCN^hi^ vessels’ decline and improved bone mass in both OVX and aging animals. These findings demonstrated the role of RBP-YBX1 in angiogenesis-dependent bone formation and provided a therapeutic approach for ameliorating osteoporosis.

## Abstract 2

<p>RNA-binding protein YBX1 promotes type-H vessel-dependent bone formation and reverses osteoporosis.</p>

## Graphical abstract

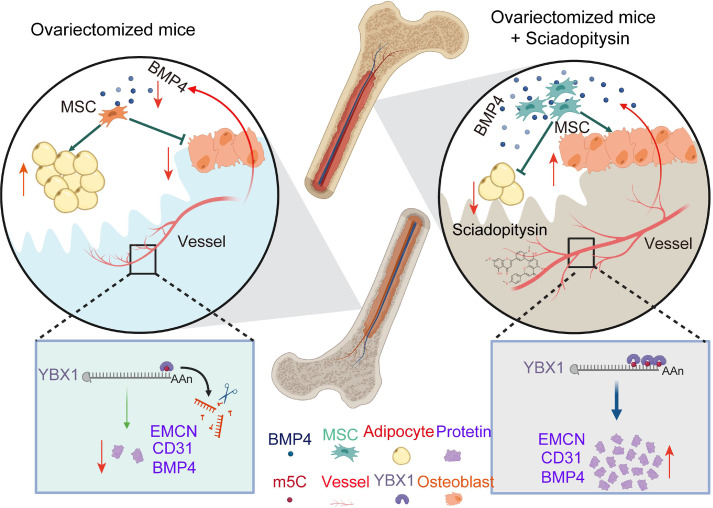

## Introduction

Skeletal tissues are rich in blood vessels that consist of type H vessels (CD31-high, endomucin-high; CD31^hi^EMCN^hi^) distributed in the metaphysis region with bud, arch, and column shapes and the highly branched type L vessels (CD31^lo^EMCN^lo^) distributed in the diaphysis region (1). The skeletal blood vessels, specifically the type H vessel subtype, control bone homeostasis, repair, and pathobiological processes, and the disruption of the vascular network is associated with the progression of bone diseases, including cancer and osteoporosis (2). Osteoporosis is a systemic bone disease characterized by decreased bone mass, impaired bone microstructure, and fracture (3). Postmenopausal osteoporosis is especially concerning because it significantly increases the fracture risk of older women. Important reports have previously been published on the fact that bone vasculature can regulate bone formation (4). Blood vessel growth is affected by estrogen, which alters the energy balance in the bone microenvironment (5). Recent research has found that the enrichment of skeletal type H vessels significantly declines in aging and ovariectomy-induced (OVX-induced) mice and patients with osteoporosis (6, 7). For one thing, the decline in type H vessels contributes to reduced nutrient delivery, tissue metabolism, and the influx of calcium and phosphate; and for another, decrease in type H vessels reduces the expression of vasogenic secreted proteins and blocks the crosstalk between endothelial cells and other cell types, including pre-osteoprogenitors, osteoprogenitors, osteoblasts, chondrocytes, and hematopoietic stem cells, and then impairs bone homeostasis (2, 8–10). In the skeletal system, at the vessel buds’ proximal end, the bone mesenchymal stromal cells (BMSCs) are also surrounded by the relatively straight, column-shaped type H capillaries (11). In addition, perivascular BMSCs interact with type H capillaries by secreting glioma-associated oncogene homolog 1 during bone development and defect healing (12). These findings suggested that BMSC-derived instructive signals regulate endodermal cells. However, it is currently unclear whether and how skeletal endothelial cell networks are coupled with the differentiation of BMSCs.

RNA-binding proteins (RBPs) are a large class of proteins with RNA-binding properties, chaperone RNA throughout its lifetime, and regulate RNA stability, modifications, and localization (13–15). Several key RBPs have been reported to modulate physiological and pathological angiogenesis by regulating the metabolism of mRNAs encoding angiogenic modulators (16, 17). However, the role of RBPs in type H vessel formation is unclear. The Y-box-binding protein 1 (YBX1), a member of the RBP family, is involved in RNA splicing, stability, and translation control during the RNA life cycle (18). Evidence from other studies indicates YBX1 is implicated in physiological and pathological angiogenesis by regulating the translation and stability of *Vegfa* and *Hif-1α* (17, 19–22). Due to the high expression of *Ybx1* in tumor-associated vasculature, *Ybx1* has also been studied as a therapeutic target to suppress tumor angiogenesis (23). Previously, we demonstrated that YBX1 was regarded as a splicing factor to regulate BMSC fate during aging (24). However, whether RBP-YBX1 participates in the regulation of angiogenesis of skeletal vessels and whether the *Ybx1* loss in endothelial cell lineage(s) is linked to osteoporotic disease are unclear.

In the present study, we found that endothelial cell–specific RBP-*Ybx1*–knockout mice showed decreased CD31^hi^EMCN^hi^ vessel density and osteogenic differentiation of BMSCs by disrupting the stability of CD31 and pro-osteogenesis factor bone morphogenetic protein 4 (BMP4). Administration of recombinant BMP4 restored the phenotype defects in *Ybx1*-deleted mice. Moreover, we constructed vessel-targeting nanoparticles, carrying sciadopitysin, which increased the CD31^hi^EMCN^hi^ vessels’ density and the secretion of BMP4 and promoted bone formation in OVX and aged mice. Taken together, our study identified a potential therapeutic target and an approach to treat postmenopausal and aging-related osteoporosis.

## Results

### RBP-YBX1 is substantially reduced in CD31^hi^EMCN^hi^ endothelial cells of OVX mice.

**Figure 1:**
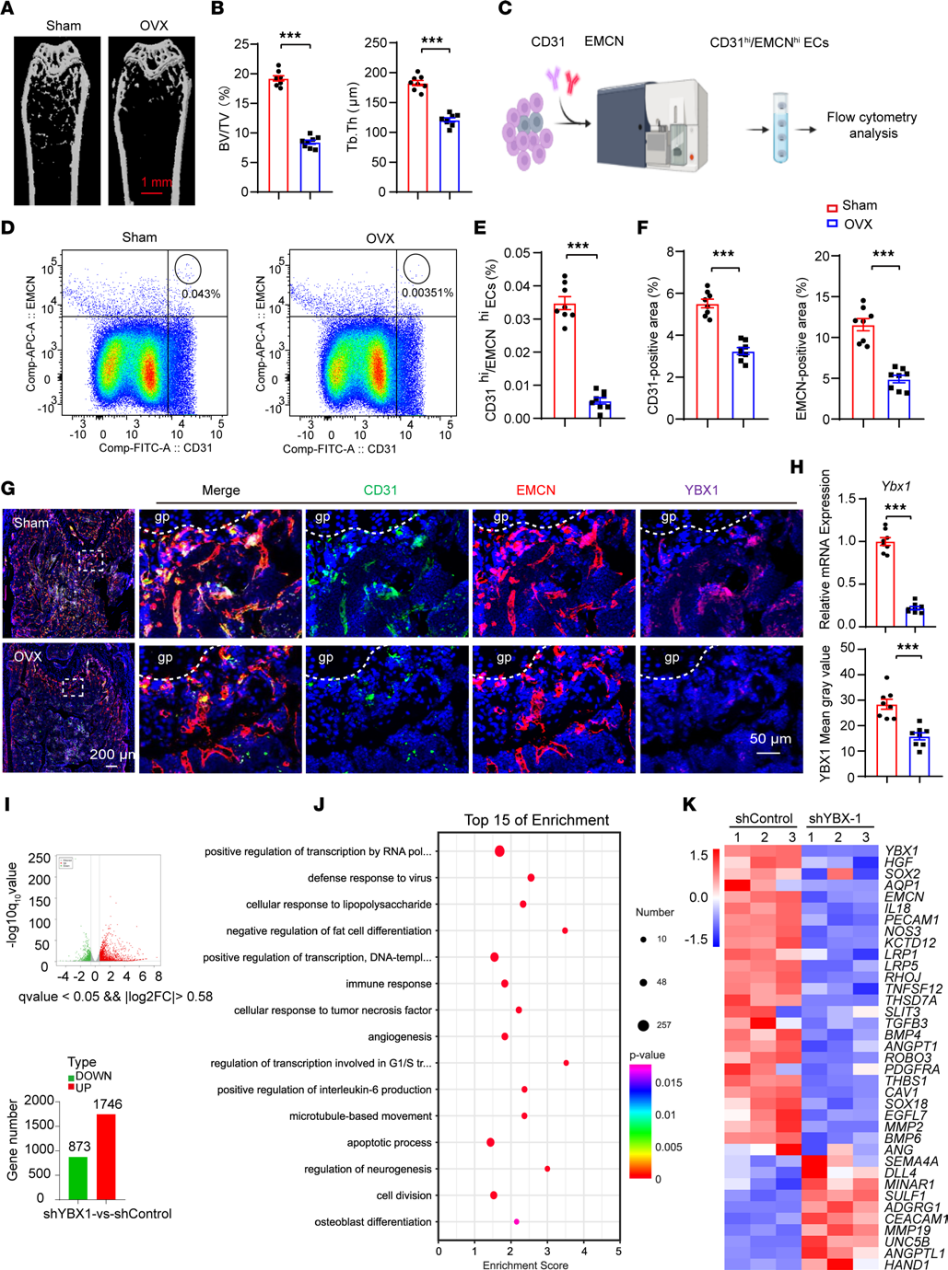
*Ybx1* is reduced in CD31^hi^EMCN^hi^ endothelial cells of OVX mice. (**A** and **B**) Representative μCT images (**A**) and quantitative μCT analysis (**B**) of femurs of 4-month-old sham-operated and OVX mice. (**C**–**E**) FACS analysis dot plot (**C** and **D**) and quantification (**E**) of CD31^hi^EMCN^hi^ endothelial cells (ECs) from sham-operated and OVX mice. (**F** and **G**) Representative images (**G**) and quantification (**F**) of CD31- (green) and EMCN- (red) stained femora from sham-operated and OVX mice. Scale bar, 200 μm and 50 μm. (**H**) RT-qPCR analysis of *Ybx1* expression in CD31^hi^EMCN^hi^ ECs (upper) and quantification of YBX1-stained (purple) femora from sham-operated and OVX mice (lower). (**I**–**K**) RNA-sequencing (RNA-Seq) data from HUVECs with knockdown of YBX1. (**I**) Differentially expressed genes in HUVECs infected with YBX1 shRNA (shYBX1) and control shRNA (shControl) adenovirus. (**J**) Gene ontology (GO) analysis revealed enrichment of biological processes among the differentially expressed genes. (**K**) Heatmap of differentially expressed genes related to angiogenesis. *n* = 8 mice in each group. *n* = 2 independent experiments. Data are shown as the mean ± SEM. ***P < 0.001 by Student’s *t* test. gp, growth plate.

To investigate *Ybx1* expression in the bone endothelium of OVX mice, we first performed OVX to investigate events under pathological conditions. Micro-computed tomography (micro-CT) and histomorphometric analysis showed a significant decrease in bone mass (bone volume over total volume; BV/TV) and thickness of trabeculae in OVX mice relative to sham controls (12-week-old) (Figure 1, A and B). Flow cytometric quantification and immunofluorescence showed a strong reduction in CD31^hi^EMCN^hi^ endothelial cells (ECs) (Figure 1, C–E) and a weak CD31 and EMCN staining (Figure 1, F and G) in OVX mice compared with the sham group. Meanwhile, we sorted CD31^hi^EMCN^hi^ ECs from OVX and control mice by flow cytometry. Real-time quantitative PCR (RT-qPCR) analysis of the collected cells revealed decreased expression of *Ybx1* in OVX mice (Figure 1H). Likewise, immunofluorescence of femur sections with specific YBX1 antibodies displayed a slightly lower *Ybx1* expression (Figure 1, G and H). These observations suggested that YBX1 protein is dynamically controlled in the OVX mouse model. Some earlier studies highlighted the potential role of *Ybx1* in blood vessel growth (22, 25). Endothelial migration and capillary tube formation were impaired in the *Ybx1*-knockout group compared with controls (Supplemental Figure 1, A–D; supplemental material available online with this article; https://doi.org/10.1172/jci.insight.172345DS1). To further identify potential pro-angiogenic factors regulated by *YBX1* in HUVECs, we performed RNA-sequencing (RNA-Seq) transcriptional profiling. Analysis of the RNA-Seq data identified 873 mRNAs with decreased translation and 1,746 mRNAs with upregulation (Figure 1I). Gene Ontology (GO) analysis demonstrated that angiogenesis- and osteogenesis-associated differentially expressed genes were enriched and comprised the top 15 GO categories in the *YBX1*-knockdown group compared with the control (Figure 1, J and K). RT-qPCR data further validated that *YBX1* knockdown decreased the levels of pro-angiogenic genes while antiangiogenic genes showed upregulation (Supplemental Figure 1E). These findings suggested a key role of *Ybx1* in angiogenesis and suggested it might be involved in the dysfunction of bone endothelium in OVX mice.

### Endothelial Ybx1 deletion impairs CD31^hi^EMCN^hi^ endothelium formation and bone formation.

**Figure 2:**
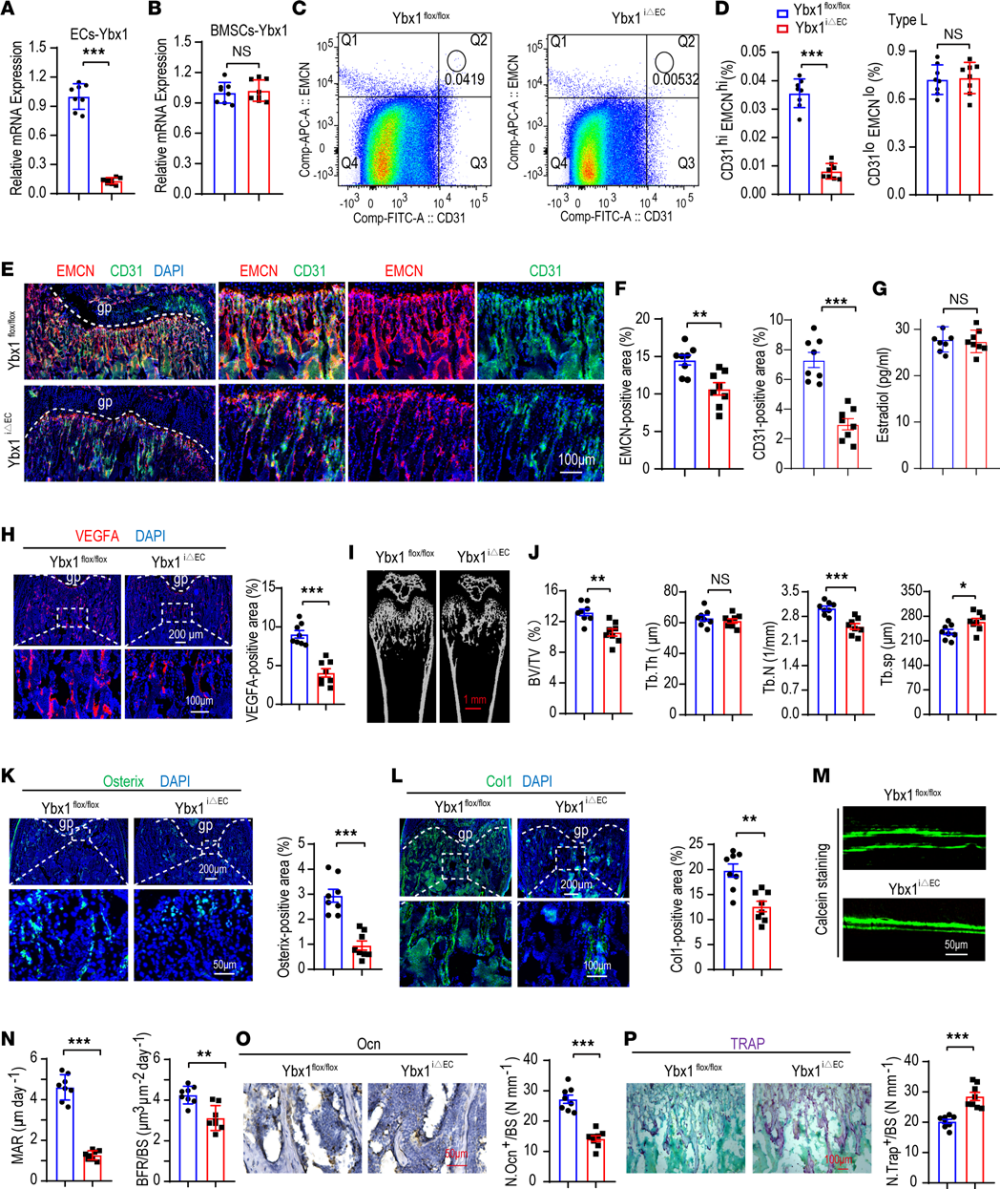
Endothelial *Ybx1* deletion impairs CD31^hi^EMCN^hi^ endothelium formation and bone formation. (**A** and **B**) RT-qPCR analysis of *Ybx1* expression in CD31^hi^EMCN^hi^ ECs (**A**) and BMSCs (**B**) from EC-specific *Ybx1*-knockout female mice (*Ybx1*^iΔEC^) and their littermate controls (*Ybx1*^fl/fl^). (**C**) FACS analysis dot plot of CD31^hi^EMCN^hi^ ECs in each group. (**D**) Quantification of type H (left) and L (right) ECs from in each group. (**E** and **F**) Representative images (**E**) and quantitation (**F**) of CD31 (green) and EMCN (red) immunostained, 4-week-old *Ybx1*^iΔEC^ and *Ybx1*^fl/fl^ femora. (**G**) ELISA analysis of estradiol levels in each group. (**H**) Representative images (left) and quantitation (right) of VEGFA (red) immunostained in each group. (**I** and **J**) Representative μCT images (**I**) and quantitative μCT analysis (**J**) of trabecular bone microarchitecture of 4-week-old *Ybx1*^iΔEC^ and *Ybx1*^fl/fl^ mice. (**K**) Representative images (left) and quantitation (right) of Osterix^+^ (green) immunostained in each group. (**L**) Representative images (left) and quantitation (right) of COL1 (green) immunostained in each group. (**M** and **N**) Representative fluorescence images (**M**) and quantification (**N**) of bone histomorphometric parameters (MAR and BFR/BS) in each group. (**O**) Immunohistochemical staining (left) and quantification (right) of Ocn^+^ cells (brown) in trabecular bone surfaces of each group. (**P**) Representative images (left) and quantification (right) of TRAP^+^ cells in trabecular bone surfaces of each group. *n* = 8 mice in each group. *n* = 2 independent experiments. Data are shown as the mean ± SEM. Scale bar, 50 μm and 100 μm. *P < 0.05; **P < 0.01; ***P < 0.001 by Student’s *t* test. Tb. BV/TV, trabecular bone volume per tissue volume; Tb. N, trabecular number; Tb. Sp, trabecular separation; Tb. Th, trabecular thickness; MAR, mineral apposition rate; BFR/BS, bone formation rate.

To further unveil the role of *Ybx1* in bone endothelium, *Ybx1*-mutant mice with EC-specific ablation of *Ybx1* (*Ybx1*^iΔEC^) were generated by combining *loxP*-flanked *Ybx1* alleles (*Ybx1*^fl/fl^) with Cdh5-Cre transgenics. RT-qPCR analysis of FACS-sorted femur ECs showed a significant knockout efficiency of *Ybx1* in *Ybx1*^iΔEC^ mice compared with control littermates (4-week-old) (Figure 2A); however, the *Ybx1* expression level of BMSCs was not affected (Figure 2B). The type H vessels (CD31^hi^EMCN^hi^) couple angiogenesis with osteogenesis, and the decrease in richness of CD31^hi^EMCN^hi^ endothelium is closely integrated with aging-associated diseases, such as postmenopausal osteoporosis (1, 6, 26). To investigate the effect of *Ybx1* on the richness of CD31^hi^EMCN^hi^ endothelium, type H ECs were sorted and quantified by flow cytometry analysis. Our data showed that the type H ECs were significantly decreased in the *Ybx1*^iΔEC^ metaphysis compared with littermate controls (Figure 2, C and D). Immunofluorescence analysis of *Ybx1*^iΔEC^ mutants revealed striking vascular defects. As shown in the experiment, the type H vessel density was strongly reduced, and column/arch patterning as well as filopodia extension were disrupted in *Ybx1*^iΔEC^ mutant metaphysis (Figure 2, E and F). The phenotype was further supported by VEGFA immunostaining, which is a crucial regulator of both normal and pathological angiogenesis secreted by ECs, perivascular cells, and mature/hypertrophic chondrocytes (27, 28) (Figure 2H). This finding is consistent with previous reports demonstrating that *Ybx1* promotes angiogenesis by upregulation of *Vegfa* (29). Estrogen deficiency is the main trigger for postmenopausal osteoporosis. In order to verify whether the disrupted bone endothelium in *Ybx1*^iΔEC^ mutant mice is driven by estrogen, we detected the serum estradiol level. ELISA analysis showed that serum estradiol levels were not changed in the *Ybx1*^iΔEC^ group compared to littermate controls (Figure 2G). Disrupted bone endothelium is accompanied by bone mass loss (1). We investigated whether loss of *Ybx1* influences bone mass in vivo. micro-CT and histomorphometric analysis showed *Ybx1*^iΔEC^ mice had a severe osteoporosis phenotype, including significantly lower trabecular bone mass and density (that is, BV/TV), trabecular thickness (Tb. Th), and trabecular number (Tb. N) and expanded trabecular separation (Tb. Sp) (Figure 2, I and J). Osterix^+^ osteoprogenitors decrease with age and have been identified to be responsible for bone loss and bone fracture (30). As expected, a remarkable decline in osteoprogenitors, cocoupling with bone vessels, was seen in 4-week-old *Ybx1*^iΔEC^ mice (Figure 2K). Likewise, collagen type 1α^+^ osteoblastic cells were reduced significantly (Figure 2L). Calcein double labeling revealed that *Ybx1*^iΔEC^ mice had significantly lower cortical bone mineral apposition rate (MAR) and bone formation rate (BFR) compared with those of their littermate controls (Figure 2, M and N). In addition, Osteocalcin (Ocn) immunostaining showed *Ybx1* mutants had fewer mature osteoblasts (Figure 2O) whereas with appreciable changes in bone-resorbing osteoclasts compared with littermate controls (Figure 2P). Thus, these findings demonstrate that endothelial *Ybx1* deletion elicits impairment of type H endothelium formation and bone formation, whereas it activates bone resorption.

### Adeno-associated virus 2/9. sup-Tie1-Ybx1 treatment alleviates OVX-induced bone loss.

**Figure 3:**
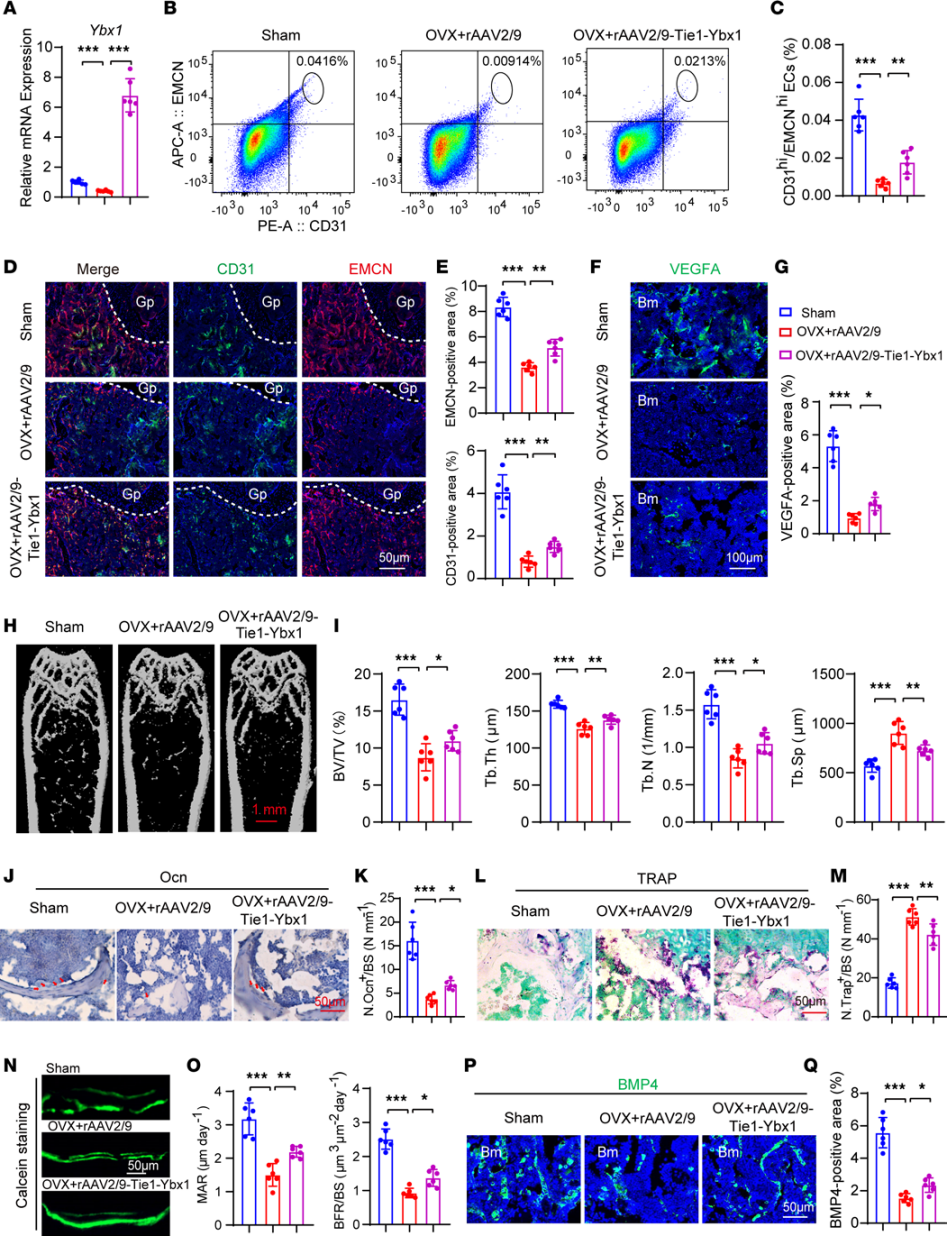
AAV2/9. sup-*Tie1*-*Ybx1* treatment alleviates OVX-induced bone loss. (**A**) RT-qPCR analysis of *Ybx1* expression in CD31^hi^EMCN^hi^ ECs from sham, OVX, and OVX mice injected with AAV2/9. sup-*Tie1*-*Ybx1*. (**B** and **C**) FACS analysis dot plot (**B**) and quantification (**C**) of CD31^hi^EMCN^hi^ ECs from each group. (**D** and **E**) Representative images (**D**) and quantitation (**E**) of CD31 (green) and EMCN (red) immunostaining in each group. Scale bar, 50 μm. (**F** and **G**) Representative images (**F**) and quantitation (**G**) of VEGFA (green) immunostaining in each group. Scale bar, 100 μm. (**H** and **I**) Representative μCT images (**H**) and quantitative μCT analysis (**I**) of trabecular bone microarchitecture of each group. Scale bar, 1 mm. (**J** and **K**) Immunohistochemical staining (**J**) and quantification (**K**) of Ocn^+^ cells (brown) in trabecular bone surfaces of each group. Scale bar, 50 μm. (**L** and **M**) Representative images (**L**) of TRAP staining and quantification (**M**) of TRAP^+^ cells in trabecular bone surfaces of each group. Scale bar, 50 μm. (**N** and **O**) Representative images (**N**) and quantification (**O**) of calcein double labeling in femora of each group. Scale bar, 50 μm. (**P** and **Q**) Representative images (**P**) and quantitation (**Q**) of BMP4 (green) immunostaining in femora of each group. Scale bar, 50 μm. *n* = 6 mice in each group. *n* = 2 independent experiments. Data are shown as the mean ± SEM. *P < 0.05; **P < 0.01; ***P < 0.001 by 1-way ANOVA.

As we reported, the loss of *Ybx1* in type H vessels is supported as a main reason for the defect of bone endothelium and bone formation. Importantly, in a gain-of-function assay, we showed that overexpression of *YBX1* promoted capillary tube formation and endothelial migration in a concentration-dependent manner (Supplemental Figure 2, A–C). Next, we conducted an in vivo experiment to evaluate the effect of EC-specific *Ybx1* overexpression on OVX-induced bone loss. OVX mouse models were generated by cutting the ovaries from 4-week-old female mice, and sham-operation mice only received non-OVX surgery. Recombinant adeno-associated virus (rAAV) 2/9 sup-Tie1-*Ybx1*-GFP was injected into mice by tail vein injection at 4 weeks postsurgery. To validate the rAAV2/9-*Tie1*-*Ybx1*-GFP virus’s effectiveness and whether it specifically targets ECs, the femurs of mice injected with rAAV2/9-*Tie1*-*Ybx1*-GFP virus were collected and then immunostained with specific GFP and CD31 and EMCN antibodies. The result showed that the red fluorescence of EMCN/CD31 and the green fluorescence of GFP greatly overlapped by analyzing the colocalization, which indicates that rAAV2/9 virus expression of GFP will specifically target ECs (Supplemental Figure 2, D and E). Meanwhile, the femurs and tibias of mice injected with rAAV2/9-*Tie1*-*Ybx1*-GFP virus or rAAV2/9-*Tie1* control virus were collected at 8 weeks postsurgery. RT-qPCR analysis of femur ECs sorted from *Ybx1*-overexpressing (*Ybx1*^iOE–EC^) mice revealed a boosted *Ybx1* mRNA level compared with control mice (Figure 3A). Flow cytometric quantification analysis validated an OVX-induced reduction of CD31^hi^EMCN^hi^ ECs, whereas EC numbers increased in *Ybx1*^iOE–EC^ mice (Figure 3, B and C). Likewise, immunostaining of femur sections showed *Ybx1*^iOE–EC^ partly rescued OVX-induced decrease in the richness of CD31^hi^EMCN^hi^ endothelium and the defect of column/arch patterning and filopodia extension (Figure 3, D and E). Also, the VEGFA density was efficiently recovered in OVX mice treated with AAV2/9 sup-*Tie1*-*Ybx1* (Figure 3, F and G). micro-CT analysis and histomorphometric analysis showed that AAV2/9 sup-*Tie1*-*Ybx1* injection markedly rescued bone loss in OVX mice (Figure 3, H and I). Likewise, the decrease of Ocn^+^ osteoblast number in femur metaphysis of OVX mice was also partly improved following AAV2/9 sup-*Tie1*-*Ybx1* treatment (Figure 3, J and K) whereas the treatment had an opposite effect on osteoclast number (Figure 3, L and M). Calcein double labeling also revealed that bone trabecular MAR and BFR were efficiently recovered in OVX mice (Figure 3, N and O). These data suggested that defective angiogenesis and osteogenesis and abnormal activation of osteoclastogenesis in OVX mice were partly reversed by treating with *Ybx1* overexpression AAV2/9 in ECs.

### YBX1 depletion leads to decreasing CD31 and EMCN stability in an m5C-dependent manner.

**Figure 4:**
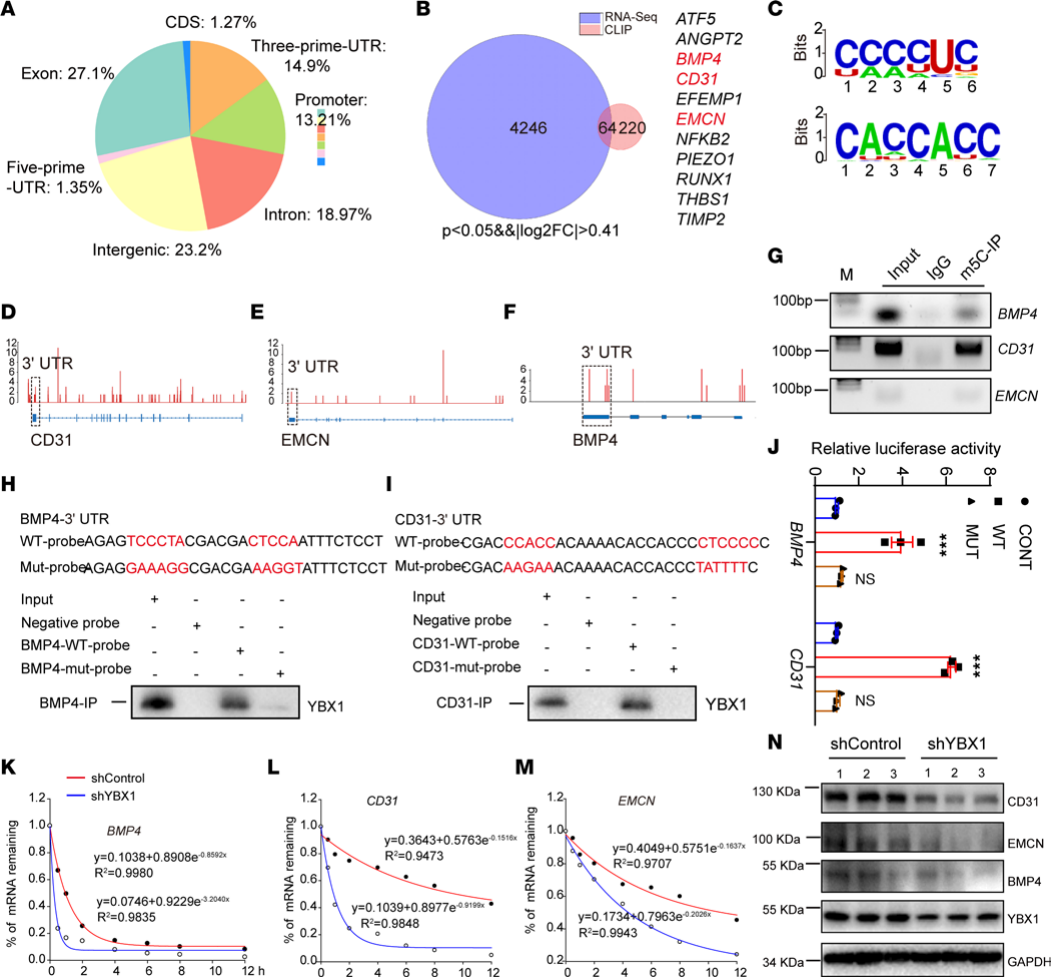
*YBX1* depletion leads to decreasing *CD31* and *EMCN* stability in an m5C-dependent manner. (**A**) Genomic distribution of *YBX1* CLIP-Seq peaks. (**B**) Venn diagram representing the overlap genes between *YBX1* CLIP-Seq targets and *YBX1*-knockdown RNA-Seq targets. (**C**) Top 2 ranked sequence motifs enriched in *YBX1* CLIP-Seq. (**D**–**F**) Genomic view of *YBX1* binding to *CD31*, *EMCN*, and *BMP4* loci. The frame area is 3′-UTR. (**G**) Semiquantitative PCR showed RBP immunoprecipitates using m5C RIP kit. (**H** and **I**) RNA pulldown analysis of binding between YBX1 protein and *CD31* (or *BMP4*)–WT (or MUT)–probe. (**J**) Relative luciferase activity of HEK293T cells transfected with different pGL-4 vectors and pCMV-YBX1. (**K**–**M**) RT-qPCR analysis of the *BMP4*, *CD31*, and *EMCN* mRNA degradation rate of HUVECs treated with shYBX1 (blue lines) or shControl (red lines). (**N**) Western blotting analysis of the relative levels of CD31, BMP4, EMCN, and YBX1 protein expression. *n* = 3 independent experiments. Data are shown as the mean ± SEM. ***P < 0.001 by 1-way ANOVA. CDS, coding sequences.

To distinguish between direct and indirect *YBX1* angiogenesis and osteogenesis-associated targets, and to investigate the detailed context-dependent rules for *YBX1* regulation, we performed cross-linking immunoprecipitation and high-throughput sequencing (CLIP-Seq). We used bowtie2 (version: 2.2.9) to map the sequenced reads to the known gene set from National Center for Biotechnology GRCh38.p14 genome database, identified 819 significant peaks using an FDR of less than 0.05, and found that most of the peaks were localized in exons (27.1%) and introns (18.97%) (Figure 4A). Notably, a sizable fraction of peaks was mapped to the 3′-UTR region (14.9%) (Figure 4A). Of the target genes assayed, we found that 64 common targets out of 4,310 *YBX1* knockdown–altered targets and out of 284 *YBX1* CLIP-Seq targets overlapped between RNA-Seq and CLIP-Seq data (Figure 4B). We were surprised to find that the most significantly enriched target genes involved angiogenesis, including pro-angiogenesis genes — *ANGPT2*, *CD31*, *EMCN*, *RUNX1*, and *THBS1* (Figure 4B). It suggested that *YBX1* affects the angiogenesis of ECs by regulating pro-angiogenesis genes’ expression. It is worth noting that the majority of the peaks were clustered in a specific region, implying that multiple *YBX1*-binding events are involved in regulating pre-mRNA splicing. *YBX1* has been reported to exhibit specific biological functions in the progression of pre-mRNA splicing and mRNA stabilization by direct RNA binding (31, 32). To test whether *YBX1* regulates pre-mRNA splicing of candidate targets, we initially calculated the alternative splicing (AS) changes in RNA-Seq data by measuring FDR and “IncLevelDifference” values. Exon skipping accounted for approximately 68% of all AS events, with the others including alternative 5′ splice site, alternative 3′ splice site, mutually exclusion exon, and retained intron in *YBX1* knockout versus control with an FDR < 0.05 and IncLevelDifference > 0.01 (Supplemental Figure 3, A and B). However, we noticed that no aberrant splicing was shown for EMCN and CD31 (Supplemental Figure 3, C–E). It suggested that *YBX1* affects angiogenesis and osteogenesis in an AS-independent way. Previous studies reported that *YBX1* regulates mRNA stability by binding to the 3′-UTR (31). So, we next focused on 3′-UTR regional clustered *YBX1*-binding events by identifying peaks above the gene-specific motifs. We first determined the known and novel overrepresented motifs in *YBX1*-binding clusters by using the Homer motif analysis software. Our data showed that about 24.78% of clusters contained the top-scored motif CCCCUC, and about 18.67% of clusters contained the motif ACCACC (Figure 4C). Obviously, most CLIP clusters were enriched in CC-rich hexamers. This is consistent with the fact that *YBX1* preferentially recognizes m5C-modified mRNAs in the 3′-UTR and plays essential roles in maternal mRNA stability (31, 33). Indeed, CLIP-Seq analysis revealed that *YBX1* had substantially higher peak distributions on the 3′-UTR of *CD31* and *EMCN* (Figure 4, D–F). To further assess whether *YBX1* binds to the 3 targets’ 3′-UTR, we performed m5C RBP immunoprecipitation (RIP) and found *YBX1* bound to *CD31* and *EMCN* 3′-UTR upon m5C RIP but not IgG immunocomplexes (Figure 4G). The result was validated by using biotin-labeling RNA pulldown analysis with a gene-specific probe containing the *BMP4* and *CD31* or mutant (MUT) 3′-UTR with a mutated CC-rich motif (m5C site) (Figure 4, H and I). We next determined the specific role of the *YBX1*-binding m5C site in regulating 3′-UTR activation. The *CD31* WT 3′-UTR and MUT 3′-UTR were constructed into pGL-4 vector and cotransfected with pCMV-YBX1 into HEK293T cells. As expected, *YBX1* substantially activated the luciferase activity of WT 3′-UTR but not MUT 3′-UTR (Figure 4J), providing further evidence that *YBX1* regulates mRNA levels by binding to its 3′-UTR specifically with m5C modification. Proteins that bind 3′-UTRs are associated with regulating mRNA stability, translation, and localization (34). To examine whether a loss of *YBX1* expression affects the *CD31* and *EMCN* mRNA stability, HUVECs were infected with shYBX1 and shControl for 2 days followed by treatment with actinomycin D for various times. RT-qPCR showed that *BMP4* mRNA levels were degraded faster upon actinomycin D treatment (Figure 4K), the relative *CD31* mRNA half-life (9.5 hours) was reduced by 91.5% (Figure 4L), and *EMCN* mRNA half-life (10.9 hours) was reduced by 60.5% (Figure 4M) in shYBX1-expressing HUVECs compared with the shControl group. Furthermore, *YBX1* knockdown markedly reduced CD31 and EMCN protein expression (Figure 4N). Therefore, we uncovered that deletion of *YBX1* impaired angiogenesis by disrupting *CD31* and *EMCN* mRNA stability in an m5C-dependent manner.

### BMP4 secretion from bone vessel restores bone formation by affecting BMSCsʼ osteogenic differentiation.

**Figure 5:**
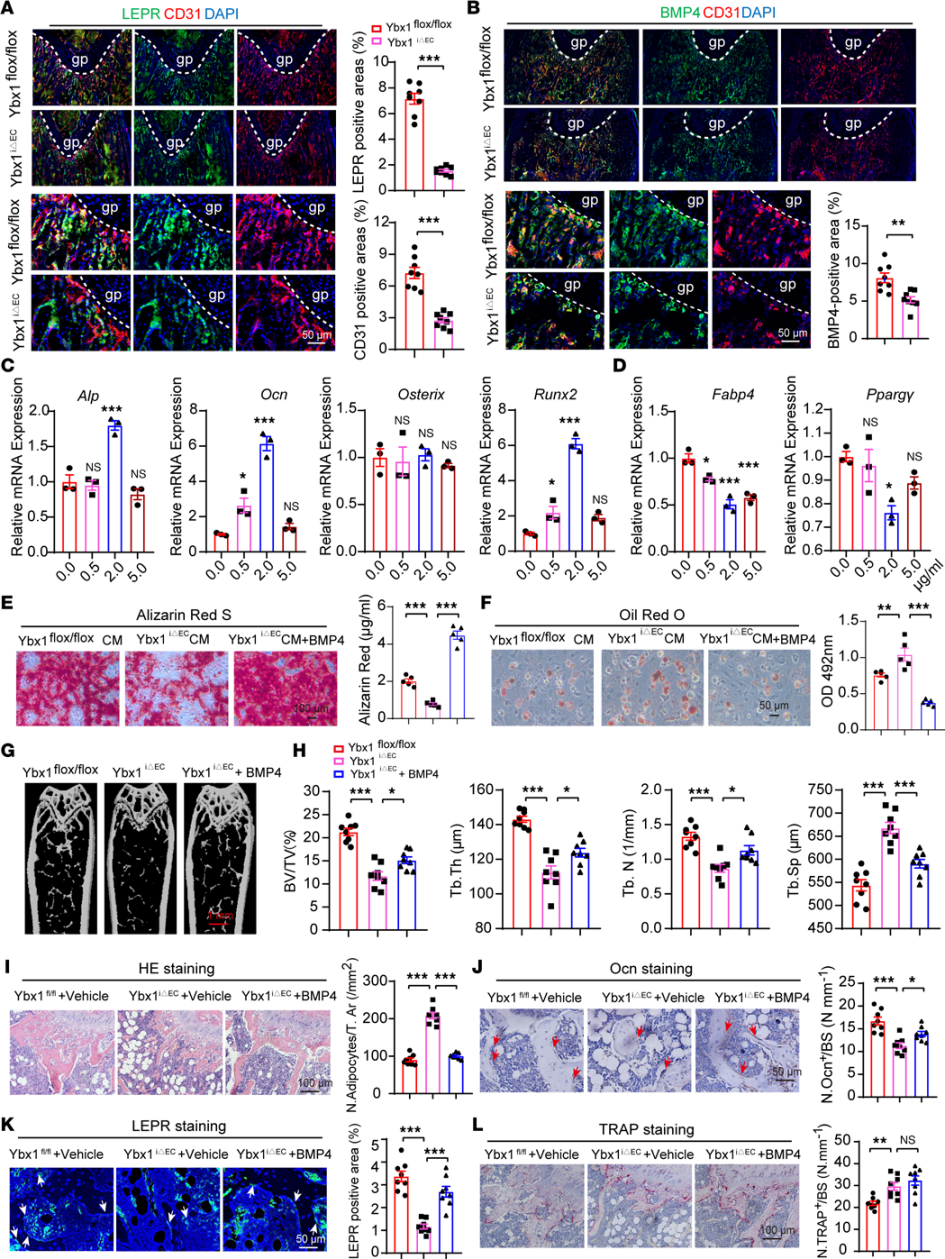
BMP4 recombinant protein restores bone formation by affecting BMSCs’ osteogenic differentiation. (**A**) Representative images and quantitation of LepR (green) and CD31 (red) immunostaining of femora. (**B**) Representative images and quantitation of BMP4 (green) immunostained femora. (**C** and **D**) RT-qPCR analysis of *Alp*, *Ocn*, *Osterix*, *Runx2*, *Fabp4*, and *Ppargγ* expression levels in BMSCs treated with different concentrations of BMP4 recombinant protein. *n* = 3 independent experiments. (**E** and **F**) Representative images and quantification of alizarin red S staining (**E**) and Oil Red O staining (**F**) of BMSCs treated with conditioned medium from primary ECs of *Ybx1*^iΔEC^ and *Ybx1*^fl/fl^ mice, with or without BMP4 recombinant protein in it. *n* = 5 independent experiments. (**G** and **H**) Representative μCT images (**G**) and quantitative μCT analysis (**H**) of trabecular bone microarchitecture of femora. (**I**–**L**) Representative images and quantification of H&E staining (**I**), Ocn immunohistochemical staining (**J**), LepR immunostaining (**K**), and TRAP staining (**L**) in trabecular bone surfaces of femora. Arrowheads indicate positive cells. Femora were obtained from 3 groups of mice, *Ybx1*^fl/fl^, *Ybx1*^iΔEC^, and *Ybx1*^iΔEC^, injected with BMP4 recombinant protein. *n* = 8 mice in each group. Scale bar, 50 μm and 100 μm. Data are shown as the mean ± SEM. *P < 0.05; **P < 0.01; ***P < 0.001 by Student’s *t* test (**A** and **B**) and 1-way ANOVA (**C**–**L**).

At the growth plate proximal end, type H vessels are strongly associated with perivascular BMSCs and osteoprogenitor cells in the metaphysis (35). In addition, an impaired functioning of type H vessels eliminated the promoting effect on BMSCs’ proliferation (36), which indicates type H vessels may be involved in affecting BMSCs’ function. To investigate whether EC-specific deletion of *Ybx1* affects BMSCs’ function and understand how EC-specific deletion of *Ybx1* affects bone formation, we harvested medium supernatant from primary bone marrow–derived (BM-derived) ECs of *Ybx1*^iΔEC^ and control mice and used the collected supernatant to culture primary BMSCs. The medium supernatant of primary ECs from the control mice displayed an enhanced ability to induce osteogenic differentiation of BMSCs, whereas it inhibited adipogenic differentiation in comparison with the basic medium. In contrast, the effects were abolished in the *Ybx1*^iΔEC^ group. (Supplemental Figure 4, A–C). Co-immunostaining of CD31 and leptin receptor (LepR) demonstrated that LepR-positive BMSCs were heavily surrounded by bone ECs, and the numbers of CD31 vessels, as well as accompanying LepR-positive BMSCs, were dramatically reduced in the *Ybx1*^iΔEC^ mice compared with their littermate control (Figure 5A). Our results suggested that bone ECs’ and BMSCs’ differentiation seems to be coupled. ECs control the behavior of other cell types in the surrounding tissue by paraclinical molecular signals (10). These results demonstrated that there is a clear vessel-derived factor crosstalk between ECs and BMSCs. To identify the potential factors regulated by *Ybx1* in ECs, we screened the common targets derived from RNA-Seq and CLIP-Seq data and identified BMP4, a pro-osteogenesis factor for coupling of angiogenesis and osteogenesis in bone development (1, 37). Our previous results showed that *YBX1* binds to the *BMP4* 3′-UTR (Figure 4, D, G, and H) and regulates *BMP4* mRNA stability in an m5C-dependent manner (Figure 4, J, K, and N). Immunofluorescence staining and quantification analysis showed that *Ybx1*^iΔEC^ mice had significantly lower *Bmp4* expression and weaker CD31 staining in comparison with littermate controls (Figure 5B) whereas overexpression of YBX1 rescued BMP4 loss in OVX mice (Figure 3, P and Q). In addition, we were surprised to find that *Bmp4* was expressed in the bone endothelium, strongly indicating that BMP4 is the potential vessel-derived factor for coupling angiogenesis and BMSCs’ differentiation. We collected culture supernatant from primary ECs from *Ybx1*^iΔEC^ and control mice. The supernatant BMP4 levels were measured, and we found that the *Ybx1*^iΔEC^ group had lower BMP4 levels (Supplemental Figure 5C). To determine whether *Bmp4* is involved in the regulation of BMSCs’ differentiation by bone ECs in vitro, we treated primary BMSCs with BMP4 recombinant protein and induced osteogenic and adipogenic differentiation. RT-qPCR analysis showed that treatment of BMSCs with BMP4 led to upregulation of alkaline phosphatase (*Alp*), *Ocn*, and runt related transcription factor 2 (*Runx2*) transcripts and downregulation of fatty acid binding protein 4 (*Fabp4*) and peroxisome proliferator–activated receptor γ (*Ppargγ*) in a concentration-dependent manner (Figure 5, C and D). Meanwhile, BMSCs showed diminished osteogenesis and enhanced lipogenesis after treatment with medium from primary BM-derived ECs of *Ybx1*^iΔEC^ mice. However, this effect was reversed by the addition of BMP4 recombinant protein to the medium (Figure 5, E and F). This is consistent with the recombinant protein’s known role in bone formation in that *Bmp4* promoted primary BMSCs’ differentiation into osteoblasts in vitro (38). We next assessed whether administration of BMP4 restored the formation of trabeculae in EC-specific *Ybx1*^iΔEC^ mice in vivo. BMP4 recombinant protein was injected daily into *Ybx1*^iΔEC^ mice (3 μg/g) for 4 weeks before analysis at day 28. In sharp contrast, administration of BMP4 recombinant protein rescued the structural alterations seen in the *Ybx1*^iΔEC^ metaphysis and bone vasculature (Figure 5, G and H), reduced bone marrow fat accumulation (Figure 5I), enabled trabeculae formation (Figure 5J), and improved the normalized number of LepR-positive BMSCs (Figure 5K) but without detectable change in tartrate resistant acid phosphatase–positive (TRAP-positive) osteoclasts (Figure 5L). Our data demonstrate that bone vessel–derived factor BMP4 is actively involved in the coupling effect of endothelial *Ybx1* on bone angiogenesis and osteogenesis.

### Polyethylene glycol–poly (lactic-co-glycolic acid) nanoparticles, carriers of sciadopitysin, act as YBX1 agonists and enhance angiogenesis-dependent bone formation in OVX mice.

**Figure 6:**
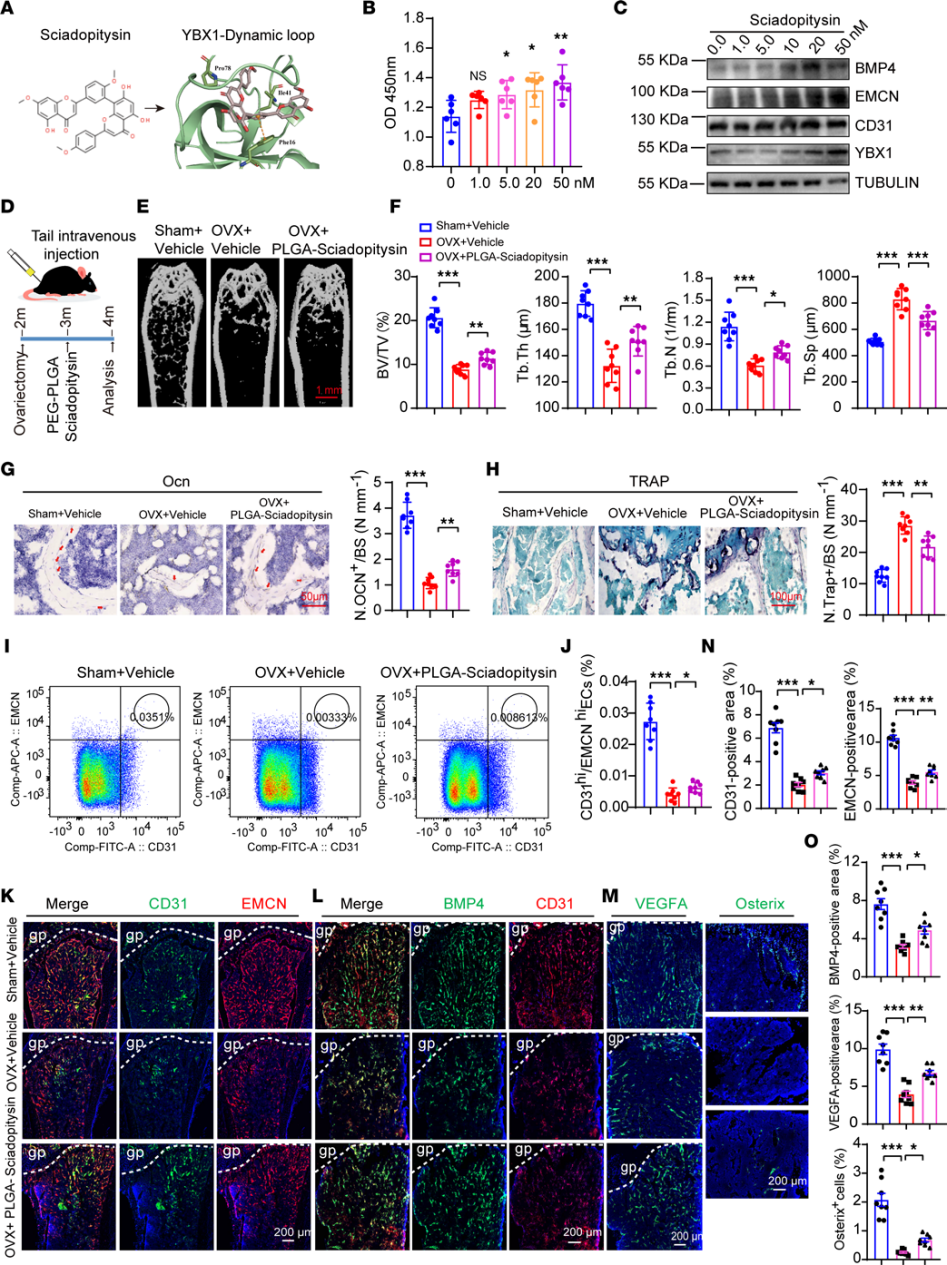
Polyethylene glycol–poly (lactic-co-glycolic acid) nanoparticles carrying sciadopitysin enhance angiogenesis-dependent bone formation in OVX mice. (**A**) The molecular structure of sciadopitysin (left) and the optimized binding modes with mouse YBX1 (right). (**B**) CCK8 assay analysis of the HUVECsʼ viability after treating with different concentrations of sciadopitysin. *n* = 6 independent experiments. (**C**) Western blotting analysis of BMP4, EMCN, CD31, and YBX1 protein levels in HUVECs treated with sciadopitysin. *n* = 3 independent experiments. (**D**) Schematic diagram of treating OVX mice with sciadopitysin. (**E** and **F**) Representative μCT images (**E**) and quantitative μCT analysis (**F**) of trabecular bone microarchitecture of femora. (**G** and **H**) Immunohistochemical staining and quantification of Ocn staining and TRAP staining in trabecular bone surfaces of femora. Scale bar, 50 μm and 100 μm. (**I** and **J**) FACS analysis dot plot (**I**) and quantification (**J**) of CD31^hi^EMCN^hi^ ECs from femora and tibia. (**K**–**O**) Representative images and quantitation (**N** and **O**) of CD31- (green) and EMCN- (red) immunostained (**K**), BMP4- (green) and CD31- (red) immunostained (**L**), and VEGFA- (green) and Osterix^+^- (green) immunostained (**M**) tibia. *n* = 8 mice in each group. Scale bar, 200 μm. *n* = 2 independent experiments. Data are shown as the mean ± SEM. *P < 0.05; **P < 0.01; ***P < 0.001 by 1-way ANOVA.

Evidence to date indicates that the enrichment of type H vessels is significantly lower in the bone of OVX mice (6). Notably, our results demonstrated *Ybx1* expression was slightly reduced, which correlates with the postmenopausal osteoporosis-related loss of type H vessels and bone mass. According to our findings above, we reasoned that enhancing the expression of *Ybx1* may be a potential therapeutic strategy to alleviate OVX-induced type H vessel richness reduction and bone mass loss. Our previous studies showed that some small molecular compounds that are naturally antiinflammatory and antioxidative, including theaflavin 3-gallate (TF2A), eriocitrin, sciadopitysin, bilobetin, and isoginkgetin, target YBX1 by binding to its pocket-like structure (Supplemental Figure 6, A–D; Figure 6A; and ref. 39). A cell proliferation assay was performed in ECs to analyze the effect of 5 natural compounds on HUVECs’ cell viability. The results showed that TF2A and sciadopitysin enhanced HUVECs’ cell viability while isoginkgetin markedly inhibited cell activity. There was no detectable change after eriocitrin and bilobetin treatment (Supplemental Figure 6E). A tube formation assay with matrigel revealed that sciadopitysin treatment resulted in the substantial increase of total branching points relative to vehicle control, while TF2A and eriocitrin treatment caused no alteration (Supplemental Figure 6, F and G). In addition, RT-qPCR analysis revealed that sciadopitysin treatment resulted in modestly higher transcript levels of *BMP4*, *CD31*, *EMCN*, and *VEGFA* but displayed no effect on *YBX1* transcript levels. Likewise, there was no detectable change after TF2A and eriocitrin treatment (Supplemental Figure 6H). To further verify the above findings, similar experiments were performed in HUVECs with different concentrations of sciadopitysin. We also discovered that sciadopitysin treatment of HUVECs increased EC migration (Supplemental Figure 6, I and J), tube formation (Supplemental Figure 6, K and L), and cell viability in a concentration-dependent manner (Figure 6B). Moreover, Western blotting results showed that treatment of HUVECs with sciadopitysin led to upregulation of *YBX1* and of expression of its target genes *CD31*, *EMCN*, and *BMP4* (Figure 6C). This is consistent with the demonstration that sciadopitysin enhances *YBX1* stability by blocking the ubiquitination-mediated protein degradation pathway (24). Next, we tested whether pharmacological administration of sciadopitysin ameliorated the reduction of CD31^hi^EMCN^hi^ ECs and osteogenesis in OVX mice. With a diameter of 20 to 80 nm and the ability to avoid immune recognition and clearance, biocompatible, biodegradable nanoparticles (NPs) exhibit excellent properties for carrying candidate drugs (40, 41). So, we constructed a nanocarrier by conjugating CD31 antibody (Alexa Fluor 488 conjugate) to NH2–polyethylene glycol (NH2-PEG) modified on poly (lactic-co-glycolic acid) (PLGA) NH2-PEG-PLGA NPs to target ECs (Supplemental Figure 7A). To directly assess the efficiency and specificity of CD31-NH2-PEG-PLGA NPs delivering sciadopitysin into ECs in vitro, HUVECs were incubated with CD31-labeled NPs for 12 hours and then stained with DAPI. We discovered a significant accumulation of green fluorescent NPs in HUVECs, indicating that CD31-labeled NPs could efficiently transport sciadopitysin to ECs (Supplemental Figure 7B). We next modified CD31-labeled NPs with Cy7 dye and sought to analyze the biodistribution of the NPs. As shown, the Cy7 signal was readily detected in the heart, lung, spleen, liver, kidneys, and hind limbs, which validated the efficient distribution of CD31-labeled NPs to the skeletal and nonskeletal organs (Supplemental Figure 7C).

Next, CD31-labeled NPs were injected in OVX mice at day 30 postsurgery (10 mg/kg) by tail vein injection 3 times a week for 30 consecutive days. As predicted, OVX mice treated with CD31-labeled NPs had significantly greater bone mass, osteoblast number, and osteoprogenitor number (Figure 6, E–G, M, and O). Meanwhile, the osteoclast number was significantly reduced compared with OVX control mice (not treated with CD31-labeled NPs) (Figure 6H). Cortical bone MAR and BFR were efficiently recovered (Supplemental Figure 6, M and N). Moreover, flow cytometric quantification at 30 days postirradiation showed type H ECs were significantly increased in comparison with OVX control mice (Figure 6, I and J). Furthermore, 30 days of CD31-labeled NP treatment led to substantial expansion of CD31^hi^EMCN^hi^ ECs in OVX mice (Figure 6, K and N). Likewise, VEGFA and BMP4 fluorescence intensities were significantly increased (Figure 6, L, M, and O). Taken together, these results give proof of the principle that CD31-labeled sciadopitysin NPs might have clinical utility to ameliorate disorders of low bone mass, such as postmenopausal osteoporosis.

### PEG-PLGA nanoparticles, carriers of sciadopitysin, act as YBX1 agonists and enhance angiogenesis-dependent bone formation in aged mice.

**Figure 7:**
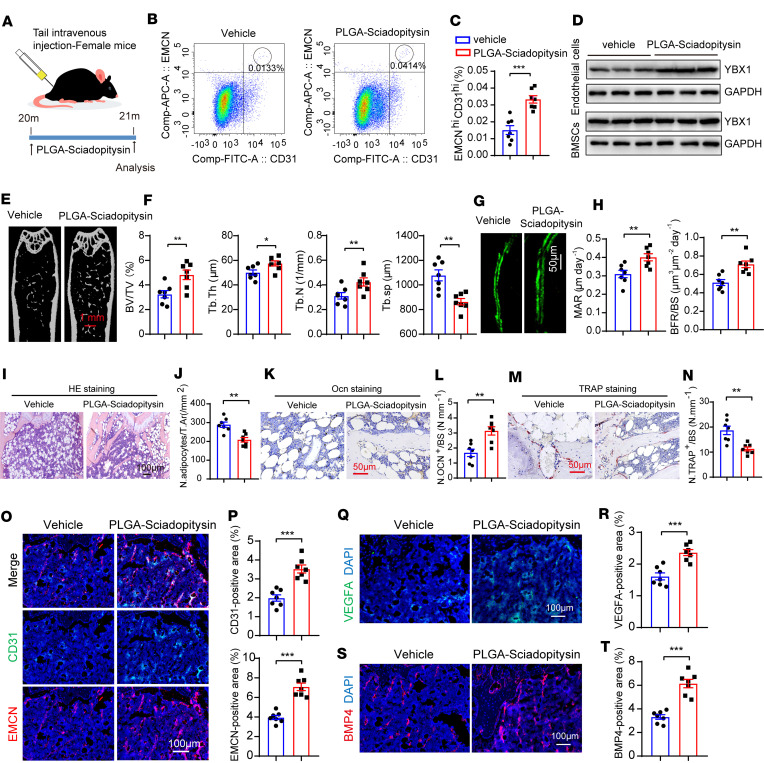
PEG-PLGA nanoparticles carrying sciadopitysin enhance angiogenesis-dependent bone formation in aged female mice. (**A**) Schematic diagram of treating aged female mice with sciadopitysin. (**B** and **C**) FACS analysis dot plot (**B**) and quantification (**C**) of CD31^hi^EMCN^hi^ ECs from femora and tibia. (**D**) Western blotting analysis of YBX1 in CD31^hi^EMCN^hi^ ECs (upper) and BMSCs (lower) from aged female mice injected with vehicle (PEG-PLGA nanoparticles) and PEG-PLGA nanoparticles carrying sciadopitysin (CD31 modified). (**E** and **F**) Representative μCT images (**E**) and quantitative μCT analysis (**F**) of trabecular bone microarchitecture of femora. (**G** and **H**) Representative images (**G**) and quantification (**H**) of calcein double labeling in femora. Scale bar 50 μm. (**I**–**N**) Representative images and quantification of H&E staining (**I** and **J**), Ocn staining (**K** and **L**), and TRAP staining (**M** and **N**) in trabecular bone surfaces. Scale bar 100 μm and 50 μm. (**O** and **P**) Representative images (**O**) and quantitation (**P**) of CD31 (green) and EMCN (red) immunostaining of tibia. (**Q** and **R**) Representative images (**Q**) and quantitation (**R**) of VEGFA-immunostained (green) tibia. (**S** and **T**) Representative images (**S**) and quantitation (**T**) of BMP4-immunostained (red) tibia. Femora and tibia were obtained from aged female mice injected with vehicle (PEG-PLGA nanoparticles) and PEG-PLGA nanoparticles carrying sciadopitysin (CD31 modified). Scale bar 100 μm. *n* = 7 mice in each group. *n* = 2 independent experiments. Data are shown as the mean ± SEM. **P < 0.01; ***P < 0.001 by Student’s *t* test.

Next, to determine the therapeutic effects of sciadopitysin on endothelium formation and bone formation in aging male and female mice, CD31-labeled NPs were injected into 20-month-old female mice (10 mg/kg) by tail vein injection 3 times a week for consecutive 30 days (Figure 7A). Flow cytometry analysis showed that the administration of CD31-labeled NPs substantially countered age-induced CD31^hi^EMCN^hi^ endothelium loss (Figure 7, B and C). Western blotting results showed that CD31-labeled NPs activated YBX1 expression in CD31^hi^EMCN^hi^ ECs but not in BMSCs (Figure 7D). micro-CT analysis and histomorphometric analysis showed that CD31-labeled NP treatment promoted bone formation (Figure 7, E–H), increased mature osteoblast numbers (Figure 7, K–L), and decreased bone marrow fat accumulation (Figure 7, I and J), as well as osteoclast numbers (Figure 7, M and N). Immunofluorescence staining analysis showed that CD31^hi^EMCN^hi^ ECs’ density (Figure 7, O and P), as well as VEGFA (Figure 7, Q and R) and BMP4 fluorescence intensities (Figure 7, S and T), were similarly improved in comparison with control mice. Next, we treated 20-month-old male mice with CD31-labeled NPs for 30 consecutive days (Supplemental Figure 8A). CD31^hi^EMCN^hi^ ECs’ density (Supplemental Figure 8, B–E), VEGFA (Supplemental Figure 8, F and G) and BMP4 fluorescence intensities (Supplemental Figure 8, H and I), and Osterix^+^ osteoprogenitor number (Supplemental Figure 8, J and K) were similarly improved in comparison with control mice. However, the osteoclast numbers did not show any noticeable changes (Supplemental Figure 8, L and M).

Taken together, these results suggested that elevating the expression of *Ybx1* by intravenous administration of sciadopitysin promoted CD31^hi^EMCN^hi^ vessel formation and stimulated new bone formation in both aged and OVX-induced osteoporosis mouse models.

## Discussion

RBPs are a huge and complicated group that is essential to posttranscriptional gene regulation. RBPs can also regulate the formation of vessels in tissues, which is lesser known but not less important (17). In the present study, we are the first to our knowledge to elucidate that RBP-*Ybx1* promotes type H vessel–dependent bone formation by regulating *CD31* and *Bmp4* stability in an m5C-dependent manner. As we know, YBX1 is a multifunctional factor that involves the processes of mRNA transcription, stabilizing, splicing, protein packaging, and translation (42, 43). One of our previous studies revealed that *Ybx1*, an AS of pre-mRNA, regulates BMSCs’ senescence and differentiation during aging (24). In fact, AS of pre-mRNA is an important posttranscriptional way of governing the process of angiogenesis by generating alternatively spliced isoforms of key pro-angiogenesis genes, including *VEGFA*, *VEGFR1*, *VEGFR2*, *NRP-1*, *FGFR*s, *Vasohibin-1*, *Vasohibin-2*, *HIF-1α*, *Angiopoietin-1*, and *Angiopoietin-2* (44). However, in our study, we did not find any significant alterations of alternatively spliced isoforms of these candidate pro-angiogenesis genes. Nevertheless, we cannot exclude the possibility that *Ybx1* regulates type H vessels’ formation by affecting pre-mRNA AS of other angiogenesis-related genes.

Another important finding in the present study was the recognition of the communication between type H vessels and differentiating BMSCs. Previous studies were focused on the coupling of type H vessels and osteoprogenitors but ignored the coupling of type H vessels and BMSCs. Like the osteoprogenitors, BMSCs are surrounded with vessels in bone tissues (Figure 5A) (2). In the context of bone formation, BMSCs are multilineage potential stem cells and are the source of new osteoblasts and their progenitors, which are essential for bone homeostasis and fracture healing (45). Studies supported that the age-related switch between osteoblast and adipocyte differentiation of BMSCs is a major factor for age-related bone loss (46, 47). Here we demonstrated that deletion of *Ybx1* in ECs decreased the expression of EC-derived *Bmp4* and then disrupted the switch between adipogenic differentiation and osteogenic differentiation of BMSCs. Our results further demonstrate that ECs are often a source of paracrine molecular signals and control the behavior of other cell types in the surrounding tissue by secreting EC-derived instructive factors (37, 48). We also propose that type H ECs mediate local proliferation of BMSCs and provide niche signals for BMSCs.

There is no doubt that *BMP4* has been implicated in the induction of osteoblast differentiation during embryonic skeletogenesis and fracture healing (37, 49, 50). Meanwhile, *BMP4*-induced commitment to the osteoblastic lineage is a prerequisite for osteoclast development, and *BMP4* depletion or treatment with a *BMP4* signaling inhibitor diminishes osteoclast differentiation (49, 51). As such, it is reasonable to assume that osteoclastogenesis is impaired in the *Ybx1*^iΔEC^ mice. However, as we show in the present manuscript, deletion of *Ybx1* in ECs instead promoted osteoclastogenesis. TRAP stain analysis in *Ybx1*^iΔEC^ mouse femur showed that a mass of osteoclasts was enriched on the bone surface (Figure 2, P and Q). This suggested that abnormally activated osteoclastic differentiation may be considered another cause for *Ybx1* deletion–induced bone loss. This result also strongly suggests the existence of other factors that promote osteoclast differentiation. Excitingly, we found mRNA levels of *Tnf*α, a crucial differentiation factor for osteoclast activity (52), were markedly increased in shYbx1 samples by analyzing the RNA-Seq data. The result was further validated by measuring *Tnfα* levels in the medium from primary BM-derived ECs of *Ybx1*^iΔEC^ and control mice (Supplemental Figure 5, A–C). In addition, the promotional effect of *Ybx1*^iΔEC^ conditioned medium on osteoclastic differentiation was counteracted by TNF-α neutralizing antibody (Supplemental Figure 5, F and G). Moreover, the serum TNF-α levels of OVX mice were decreased after treatment with AAV2/9 sup-*Tie1*-*Ybx1* (Supplemental Figure 5H). The transcription factor analysis experiments indicated that *Ybx1* may regulate the expression of interferon regulatory factor 1 and then indirectly regulate TNF-α levels (Supplemental Figure 5, D and E). However, the exact molecular mechanism underlying the different role of *Ybx1* in osteoclastic differentiation remains to be elucidated.

PLGA is one of the biodegradable polymeric NPs with desirable stained-release properties, low toxicity, and biocompatibility with tissue and cells and has been widely applied in drug delivery systems for the treatment of many diseases (53). Sciadopitysin is a natural compound isolated from the traditional Chinese herbal agent with desirable antioxidant, anti-osteoclastogenesis, and anti-Alzheimer’s disease properties (54–56). Our previous study supported that sciadopitysin enhanced the protein expression of *Ybx1* by blocking the ubiquitinated degradation pathway. However, its poor water solubility remains a clinical challenge. In this study, we examined the possibility of loading sciadopitysin into CD31-modified NPs and tested the effectiveness of treatment for the decline of type H vessels and bone mass in OVX and aging mice. In our study, we found that sciadopitysin is selectively delivered to the vasculature by CD31-modified NPs, and the defective bone angiogenesis and osteogenesis in OVX and elderly mice were successfully reversed after targeted administration of CD31-modified sciadopitysin-carrying NPs. We believe this work offers an avenue to increase the clinical utility of sciadopitysin in the treatment of osteoporotic diseases.

In conclusion, we revealed that YBX1 acts as an RBP that promotes type H vessel–dependent bone formation by coupling with BMSCs’ differentiation. The CD31-modified sciadopitysin-carrying NPs can be used to boost type H vessel formation and osteogenesis in aged and OVX mice. Our findings presented here set the groundwork for novel therapeutic approaches in the management of osteoporotic disease states by boosting angiogenesis-dependent osteogenesis.

## Methods

### Mice.

All mice we used were on a pure C57/B6 background. The Cdh5-Cre transgenic mice were purchased from The Jackson Laboratory; *loxP*-flanked *Ybx1* mice were purchased from Cyagen Biosciences. *Ybx1*^+/–^ mice were crossed with *Ybx1*^+/–^ mice to generate *Ybx1*^+/+^ (WT) and *Ybx1*^–/–^ (*Ybx1*^iΔEC^) mice. WT littermates of the same sex were used as controls. For endothelium-specific *Ybx1*-knockout experiments, 8 female mice of 3 weeks of age were used for each group. C57BL/6J female and male mice aged 20 months were bought from Charles River Animal Company. For the OVX model, 4-week-old female mice were bilaterally ovariectomized or sham-operated. A month after surgery, the OVX mice were treated with either sciadopitysin or rAAV2/9-Tie1-*Ybx1*. For sciadopitysin treatment, OVX mice were randomly divided into 2 groups and injected with sciadopitysin via the tail vein at a dose of 25 mg/kg every other day for 1 month. The sham group and control OVX group instead received vehicle reagents. For AAV treatment, OVX or sham mice were injected with 100 μL rAAV2/9 (1 × 10^12^ viral genomes/mL) expressing *Ybx1* via the tail vein for a month. Three days after the last injection, all mice were euthanized, and femurs and tibias were collected for follow-up experiments.

### rAAV2/9 virus construction and treatment.

The rAAV2/9 virus was constructed with the help of Hanbio Biotechnology Co. Briefly, the *Ybx1* CDS region was cloned and inserted into the downstream of *Tie1* promoter of pHBAAV-MCS-3flag-T2A-ZsGreen shuttle plasmids (HH20230215HNSLY-AAV001). The recombinant shuttle plasmids were identified by DNA sequencing and then cotransfected into HEK293T cells with pAAV-RC and pHelper plasmids. After transfection for 72 hours, HEK293T cells were collected and lysed, and the rAAV2/9 with Tie1 promoter was separated and purified by ultrafiltration tubes (MilliporeSigma, UFC905008). The rAAV2/9 virus titer was determined by RT-qPCR, and the virus was injected into mice through the tail vein 1 time. The primer sequences are listed in Table 1.

### Cell culture and functional assays.

The HUVECs were bought from American Type Cell Collection. The HUVECs were cultured in Endothelial Cell Medium (ECM; ScienCell, 1001) and were maintained at 37°C and 5% CO_2_ in a humidified atmosphere. ECM consists of 500 mL of basal medium, 25 mL of fetal bovine serum, 5 mL of EC growth supplement, and 5 mL of penicillin/streptomycin solution. Cell culture dishes and plates were purchased from NEST Biotechnology. For the cell viability assay, HUVECs were inoculated in a 96-well plate (1 × 10^4^ cells in 200 μL of culture medium per well) and cocultured, respectively, with natural small molecular compounds TF2A, eriocitrin, sciadopitysin, isoginkgetin, and bilobetin purchased from Target Molecule Corp in an incubator containing 5% CO_2_ at 37°C for 48 hours. After that, the CCK8 reagents (MedChemExpress, HY-K0301) were added to the cell medium (10 μL for each well) and incubated for another 4 hours. The absorbance was measured at 450 nm using an ultra-micropore plate spectrophotometer (Epoch, BioTek). The OD values were calculated from 3 independent experiments. EC migration assays were performed in 24-well plates. The treated or untreated HUVECs (1 × 10^4^) were seeded into the upper chamber with 200 μL medium, and 600 μL serum-free medium was added to the lower chamber. At 16 hours after seeding, the chambers were fixed and stained with 0.2% crystal violet (Beyotime Biotechnology, C0121). The nonmigrating cells in the upper chamber were carefully removed using cotton swabs; the migrating cells were counted with Inverted Microscope Camera System (Leica) in 5 random fields of each filter. EC tube formation assay was conducted in 48-well plates precoated with Matrigel (Corning, 354234). The treated or untreated HUVECs (2 × 10^4^) were seeded into wells and incubated for 12 hours. The tube-like structures were imaged by Inverted Microscope Camera System and calculated automatically using the ImageJ software (NIH). Type H ECs were sorted using flow cytometry. Sorted ECs were then plated on dishes coated with fibronectin and cultured in EGM-2 BulletKit (Lonza).

### Osteogenic and adipogenic differentiation assay.

BMSCs were cultured in medium containing specific ingredients as described previously and treated with BMP4 or not for 21 or 10 days (57). The medium was from primary BM-derived ECs of *Ybx1*^iΔEC^ or control mice. Cells were then stained with 2% alizarin red S (G1450, Solarbio) or Oil Red O (O0625, MilliporeSigma).

### BMP4 treatment.

BMP4 recombinant protein was produced with the help of Forevertech Biotechnologies Co., Ltd. The cultured primary BMSCs were treated with different concentrations of BMP4 for the duration of 24 hours and subsequently lysed in Trizol reagent (Accurate Biology) for RT-qPCR analysis. The control and *Ybx1*-deleted mice were treated with BMP4 recombinant protein (3 μg/g) for 28 days. Three days after the last injection, all mice were euthanized, and femurs and tibias were collected for bone analysis as described earlier (58).

### ELISA analysis.

We sorted type H ECs from *Ybx1*^iΔEC^ and control mice by flow cytometry and cultured them for 48 hours. The cell media were collected and used to detect the TNF-α or BMP4 levels by the TNFα ELISA kit (R&D Systems, MTA00B) or BMP4 ELISA kit (LM-BMP4-Mu, LAMI Bio). All ELISAs were performed according to the manufacturersʼ instructions. The mice were anesthetized with ether, and blood was collected. Serum was centrifuged for 20 minutes at 2,000*g* at room temperature, and the estradiol levels were measured using QuicKey Pro Mouse E2 ELISA Kit (E-OSEL-M0008, Elabscience) according to the instructions.

### Flow cytometry and cell sorting.

The fresh femurs and tibias were dissected, then crushed in cold Hanksʼ Balanced Salt Solution, and bone pieces were digested with 1 mg/mL type 1α collagenase (MilliporeSigma, SCR136) at 37°C for 20 minutes. After filtration and washing, the cells were counted and incubated for 45 minutes at 4°C with EMCN antibody (2114518, Invitrogen) and CD31 antibody (FAB6874G, R&D Systems), then washed and further incubated with DAPI (ab285390, Abcam). Finally, the CD31^hi^EMCN^hi^ cells were acquired and demarcated on a FACScan cytometer (BD Immunocytometry Systems). The collected and sorted CD31^hi^EMCN^hi^ cells were lysed in Trizol reagent for RT-qPCR analysis. For demarcating and sorting CD31^hi^EMCN^hi^ cells, first standard quadrant gates were set; subsequently to differentiate CD31^hi^EMCN^hi^ cells from the total double-positive cells in quadrant 2, gates were arbitrarily set at >10^4^ log Fl-1 (CD31-FITC) fluorescence and >10^4^ log Fl-4 (endomucin-APC) fluorescence. The BMSCs were isolated as described previously (47).

### Immunofluorescence and immunohistochemical staining of bone sections.

For immunofluorescence staining, freshly dissected femora were fixed in 4% paraformaldehyde solution for 24 hours, then decalcified in 0.5 M EDTA (pH 7.4) at 4°C for 10 days (4-month-old mice) or 21 days (21-month-old mice). Then the bones were incubated in cryoprotectant solution (10% sucrose and 1% polyvinylpyrrolidone) for 24 hours at 4°C. After that, bone tissues were embedded in embedding solution (8% gelatin, 2% polyvinylpyrrolidone, and 20% sucrose) and transferred to a –80°C ultra-low-temperature freezer overnight. Then, the samples were then longitudinally oriented and cut to 15 μm thickness before being stained with primary antibodies against mouse CD31 conjugated to Alexa Fluor 488 (FAB3628G, R&D Systems), CD31 (553370, BD Pharmingen), endomucin (sc-65495, Santa Cruz Biotechnology), GFP (ab290, Abcam), YBX1 (4202S, Cell Signaling Technology [CST]), VEGFA (19003-1-AP, Proteintech), COL1A (AB765P, MilliporeSigma), Osterix (ab22552, Abcam), and BMP4 (30912, Forevertech Biotechnologies Co., Ltd) overnight at 4°C. The next day, the samples were incubated with secondary antibodies conjugated with fluorescence tags at room temperature for 60 minutes: Donkey anti-Goat IgG (H+L), Alexa Fluor 488 (Thermo Fisher Scientific, A-11055), Donkey Anti-Rat IgG H&L (Alexa Fluor 647) (ab150155, Abcam), Donkey anti-Rabbit IgG (H+L) Alexa Fluor 488 (Thermo Fisher Scientific, A-21206), Donkey anti-Mouse IgG (H+L) Alexa Fluor 488 (Thermo Fisher Scientific, A-21202), and Donkey anti-Rabbit IgG (H+L) Alexa Fluor 555 (Thermo Fisher Scientific, A-31572). The fluorescence signals were captured via Zeiss fluorescence microscopy (Apotome 3). For immunohistochemical staining, the bone tissues were embedded in paraffin and cut to 4 μm. The paraffin sections were dewaxed and stained with primary antibody Ocn (M173, Takara Bio), then counterstained with Harris Hematoxylin. For histochemistry staining, the 4 μm paraffin sections were stained with H&E as described previously (59).

### RT-qPCR analysis.

Total RNA from cells was extracted using AG RNAex Pro Reagent (AG21102, Accurate Biology) and reverse-transcribed into cDNA by using the 5× Evo M-MLV RT Premix (AG11706, Accurate Biology). Amplification reactions were set up in 20 μL reaction volumes containing 2× Pro Taq HS Probe Premix (AG11704, Accurate Biology), and the relative mRNA expression level was normalized to the endogenous β-actin expressions. Primer sequences are listed in Table 1.

### RNA-Seq and AS analysis.

The total RNA was extracted and used to construct shYBX1 RNA-Seq libraries as described previously (60). The cleanReads were aligned to National Center for Biotechnology Information GRCm38.p6, to obtain the location information on the reference genome and the specific sequence characteristics for the sequenced sample by hisat2. The htseq-count software was used to obtain the number of reads in each sample compared with the protein-coding gene. Then, FPKM algorithms were used to calculate gene expression, and DESeq2 software (61) (BaseMean value was used to estimate the expression) was used to test differentially expressed genes between shYBX1 and shControl groups with *q* < 0.05 and log_2_ fold-change > 0.58. GO enrichment aims to show the biological processes affected among different groups. Heatmaps were generated to show gene expression differences in different groups. AS analysis (shYBX1) was performed by rMATS (62); splicing changes with an FDR < 0.05 or FDR > 0.1 were considered statistically significant.

### RNA stability assay.

The HUVECs were infected with shYBX1 or shControl adenoviruses. After adenovirus infection (24 hours), the cells were treated with 5 μg/mL actinomycin D (HY-17559, MedChemExpress) or DMSO and collected at the indicated time points. The cells were lysed in Trizol reagent for RT-qPCR analysis.

### Western blot analysis.

The cells were lysed in RIPA buffer with protease inhibitor for 30 minutes on ice. The lysates were centrifuged at 13,000*g* for 10 minutes at 4°C, and we transferred the cell supernatant to new tubes. Then, the cell supernatant was boiled with 6× SDS loading buffer, then separated by SDS-PAGE. Proteins were transferred to polyvinylidene difluoride membranes (IPFL00010, MilliporeSigma) and incubated with specific antibodies. Primary antibodies were CD31 (20131, Forevertech Biotechnologies Co., Ltd), EMCN (sc-65495, Santa Cruz Biotechnology), YBX1 (4202S, CST), BMP4 (P00205, Forevertech Biotechnologies Co., Ltd), and Tubulin (11224-1-AP, Proteintech). Secondary anti-mouse (Ap124p, MilliporeSigma), anti-rat (BA1058, Boster Biological Technology Co., Ltd), and anti-rabbit (Ap132p, MilliporeSigma) HRP-conjugated antibodies were subsequently applied.

### CLIP-Seq.

The cross-linking and immunoprecipitation experiments were performed with a CLIP Kit (BersinBio, Bes3014-2) according to the manufacturer’s instructions. Briefly, HUVECs were treated with 4-Thiouridine with a final concentration of 100 μM for 16 hours and irradiated with 365 nm UV-C light (0.15 J/cm^2^) to covalently cross-link proteins/nucleic acids in vitro. The cross-linked cells were lysed in cell lysis buffer with DTT and protease inhibitor for 10 minutes on ice. After centrifuging the cells at 13,000*g* for 5 minutes at 4°C, the supernatant was collected and incubated overnight at 4°C with approximately 5 μg YB1 (D2A11) rabbit mAb (Cell Signaling Technology, 9744S) or IgG (Cell Signaling Technology, 5873S) and magnetic beads (MedChemExpress). We resuspended the immunoprecipitates in IP wash buffer containing RNase T1 and incubated in a 22°C water bath for 15 minutes after washing the magnetic beads with IP wash buffer. Then, the immunoprecipitates were incubated with DNase I at 37°C for 15 minutes. After that, immunoprecipitates were digested by Proteinase K, and the bound RNA was extracted with phenol:chloroform:isoamyl alcohol according to the manufacturer’s instructions and then subjected to PCR amplification. The PCR products were used to construct libraries, and the libraries were sequenced on the Illumina NovaSeq 6000 system by Wuhan Igenebook Biotechnology Co.,Ltd. Trimmomatic (version 0.38) filtered out low-quality reads from raw reads to generate clean reads. Bowtie2 (version:2.2.9) was used to map the clean reads to the GRCh38.p14 genome, and samtools (version 1.3.1) was used to remove potential PCR duplicates. CLIP-Seq peaks were identified by Piranha (version 1.2.1) with the following parameters: “-p 0.00001 -z 100 -s -o.” Homer (version 4.9.1) was used for analysis of YBX1 binding motifs.

### m5C RIP and RNA pulldown.

The m5C RIP was performed with an m5C RIP Kit from CloudSeq Biotech Inc. (GS-ET-003). Briefly, a total of approximately 100 μg RNA was digested in 1× Fragmentation Buffer, then incubated at 70°C for 6 minutes, with the reaction stopped by adding Stop Buffer. The RNA fragments were precipitated and redissolved in RNA-free water and incubated with PGM magnetic beads coupled with m5C antibody for 60 minutes at 37°C. After that, the immunoprecipitates were washed with RLT Buffer and anhydrous ethanol 3 times. Finally, the binding RNA was eluted with RNA-free water and reverse-transcribed into cDNA with random primers (AG11706, Accurate Biology). Semiquantitative PCR was performed with BMP4, CD31, and an EMCN special primer.

For the RNA pulldown experiment, BMP4- and CD31-specific biotinylated probes were synthesized by Sangon Biotech (Shanghai) Co., Ltd. and incubated with YBX1 recombinant protein (NBP2-30101, Novus) at 4°C for 3 hours. The protein/nucleic acid complex was combined with streptomycin magnetic beads and pulled down on a magnetic stand. The beads were washed with 1× PBS 4 times and then subjected to SDS-PAGE after boiling with 1× SDS loading buffer.

### micro-CT analysis and calcein double-labeling assay.

Femurs were dissected from all the experimental groups of mice. After removing the attached soft tissue thoroughly, the femurs were fixed in 4% paraformaldehyde for 24 hours. The fixed samples were scanned using high-resolution micro-CT (Skyscan 1172, Bruker microCT). The basic parameters of the scanner included x-ray energy tube voltage of 65 kV, a current of 153 μA, and a resolution of 15 μm per pixel. After scanning, the image reconstruction software (NRecon, version 1.6, Bioz), data analysis software (CT Analyser, version 1.9, Bruker microCT), and 3-dimensional model visualization software (μCT Volume, version 2.0, Bruker microCT) were used to analyze the parameters of the distal femoral metaphyseal trabecular bone. A series of planar cross-sectional images were generated in this process. We selected 5% of femoral length below the growth plate for microarchitecture analysis. The following parameters were calculated to describe the subchondral trabecular bone microarchitecture, including trabecular BV/TV, Tb. N, Tb. Sp, and Tb. Th. The calcein double-labeling assay was performed according to a previous study (47). The mice were injected with 25 mg/kg calcein at 8 and 2 days before euthanasia. The femora were fixed in 70% ethanol and dehydrated in increasing concentrations of ethanol, and the undecalcified bones were embedded in methyl methacrylate. Serial 5 μm sections of the femur were made using a microtome. The parameters we measured were BFR/bone surface and MAR (63).

### PEG-PLGA NPs.

We dissolved 10 mg of sciadopitysin in 10 mL of acetone:chloroform (7:3) solution. We added 130 μL of 100 mg/mL of PEG-PLGA (Shanghai Ponsure Biotech, Inc) chloroform solution and 200 μL of 10 mg/mL of NH2-PEG-PLGA (Shanghai Ponsure Biotech, Inc.) chloroform solution and mixed well. Then we added another 3 mL of chloroform solution and mixed well. We evaporated the above liquid to form a thin film under reduced pressure using a rotary evaporator (N-1300D-WB, EYELA). We added 2 mL of ultrapure water to the thin film and hydrated on the rotary evaporator under a water bath at 35°C for 15 minutes. The products were sonicated, and then the free unencapsulated drug was removed by centrifugation at 3,000*g* for 5 minutes at room temperature; the supernatant was the NPs.

### TRAP staining.

For osteoclast differentiation analyses in vitro, we isolated BM monocytes (BMMs) from mouse femur bones. BMMs were seeded into 24-well plates at a concentration of 1.5 × 10^4^ cells per well. Cells were cultured in the medium with 100 ng/mL RANKL (462-TEC-010, Novus Biologicals) and 50 ng/mL M-CSF (HZ-1192, Proteintech) for 6 days as previously described (57). The medium was from primary BM-derived ECs of *Ybx1*^iΔEC^ or control mice and was treated with TNF-α neutralizing antibody (50349-RN023, Sino Biological) or not. Osteoclasts were fixed and stained using the TRAP staining kit (294-67001, FUJIFILM Wako Pure Chemical Corporation). The differentiation experiments were conducted in triplicate.

### Statistics.

Statistical analyses were performed with SPSS 20.0. The data are presented as mean ± SEM. Two-tailed Student’s *t* test or 1-way ANOVA test with Tukey’s multiple comparison test was used to assess statistical significance. *P* < 0.05 was used to define statistical significance.

### Study approval.

All the mice were bred under specific pathogen–free conditions at Laboratory Animal Research Center at Central South University under protocols approved by the Medical Ethics Committee of Xiangya Hospital of Central South.

### Data availability.

Data that support the findings of this study are available in the Supporting Data Values file. The accession number for RNA-Seq data reported in this paper, for HUVECs transfected with shControl and shYbx1, is National Center for Biotechnology Information SRA: PRJNA929902, https://www.ncbi.nlm.nih.gov/bioproject/PRJNA929902/ The accession number for YBX1 CLIP-Seq data reported in this paper is National Center for Biotechnology Information SRA: PRJNA929284, https://www.ncbi.nlm.nih.gov/bioproject/PRJNA929284/

## Author contributions

YJL generated data and drafted the manuscript. QG and MSY analyzed results and revised the manuscript. GPC and WFX supervised the experiments and cowrote the manuscript. SD designed the experiments and revised the manuscript. YX designed the experiments, curated data, acquired funding, and revised the manuscript.

## Supplementary Material

Supplemental data

Unedited blot and gel images

Supporting data values

## Figures and Tables

**Table 1:**
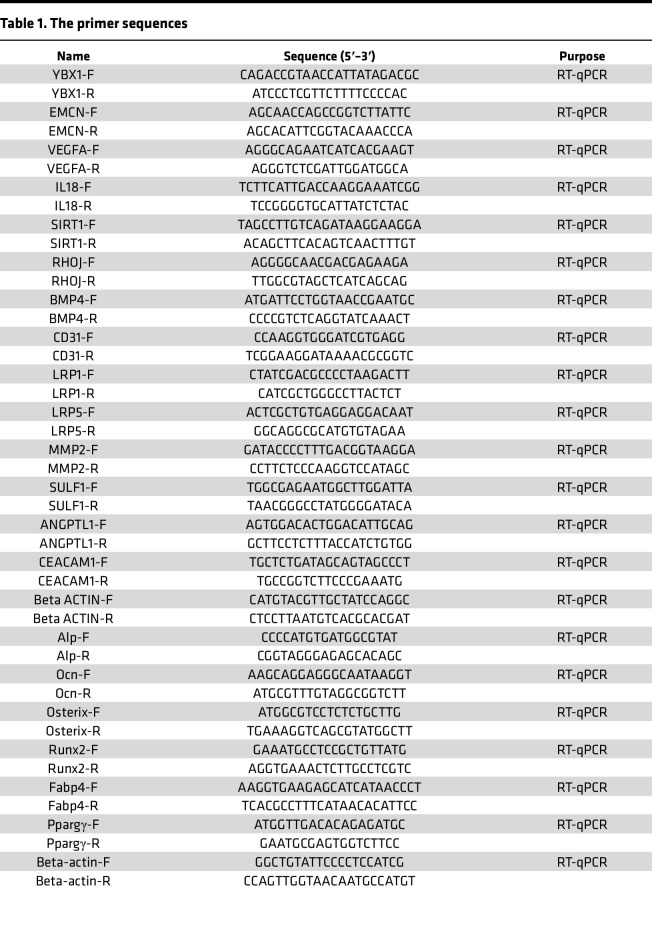
The primer sequences

## Version 1 02/22/2024

Electronic publication
