# Supplementary material for: YBX1 promotes type H vessel–dependent bone formation in an m5C-dependent manner
Source: JCI Insight. 2024 Feb 22;9(4):e172345. doi: 10.1172/jci.insight.172345 (PMC11143935; doi:10.1172/jci.insight.172345)
Supplement: Supplemental data [file jciinsight-9-172345-s057.pdf]

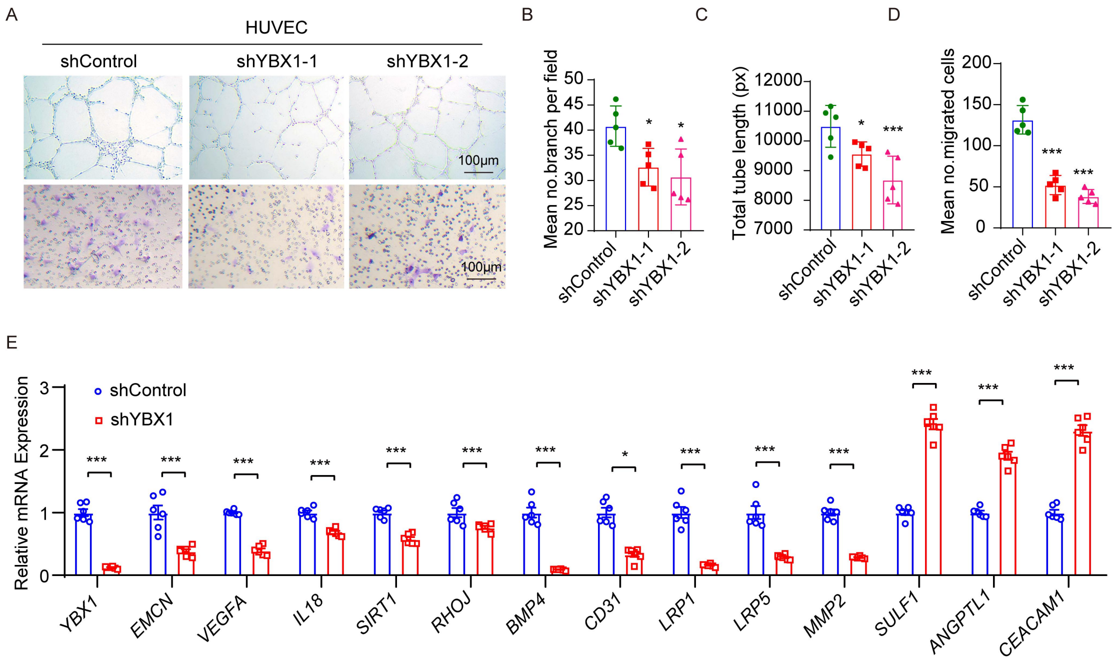

Supplemental Figure 1 Slicing of *YBX1* damaged angiogenesis of HUVECs. A-D. Representative images (A) and relative quantification (B-D) of tube branch numbers of a Matrigel tube formation assay (up panel) and a transwell migration assay (bottom panel). n = 5 independent experiments. E. RT-qPCR analysis of the expression of angiogenesis-associated genes in HUVECs infected with shYBX1 adenovirus or control adenovirus. n = 3 independent experiments. Data are shown as the mean ± SEM. *, P < 0.05; **, P < 0.01; ***, P < 0.001 by one-way ANOVA (B-D) or Student's t test (E).

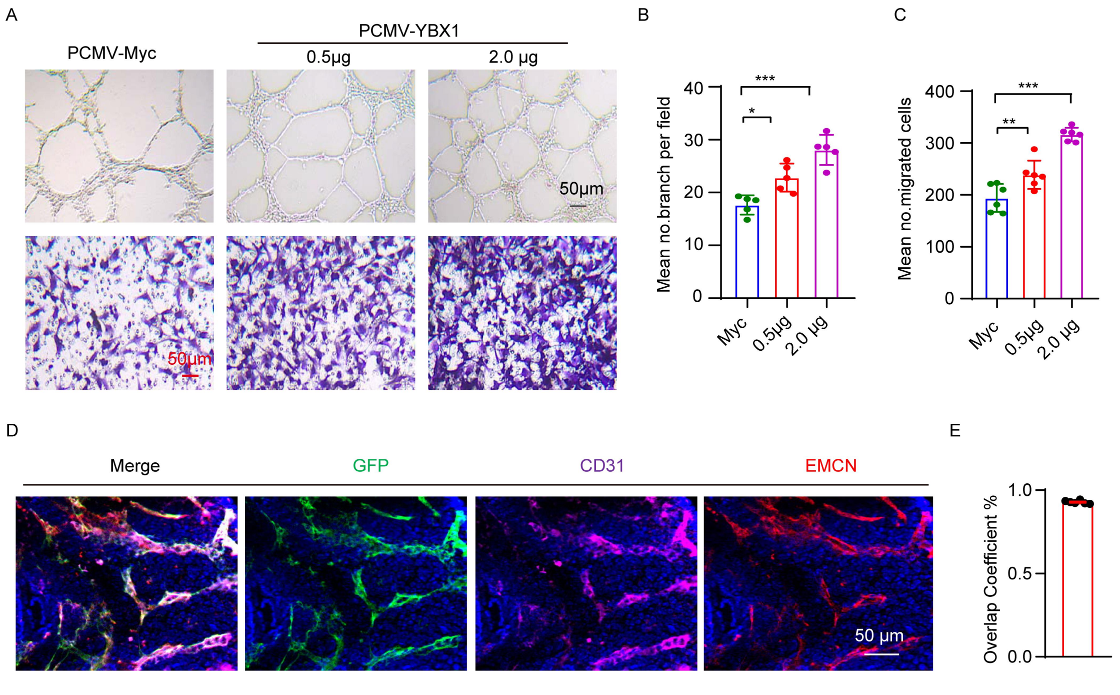

Supplemental Figure 2 Over-expression of *YBX1* enhanced angiogenesis of HUVECs. A-C. Representative images (A) and relative quantification (B-C) of tube branch numbers of a Matrigel tube formation assay (up panel) and a transwell migration assay (bottom panel). n = 5 independent experiments. D-E. Representative images (D) of GFP (green), CD31 (purple) and EMCN (red) immunostaining and overlap coefficient analysis (E) of GFP, CD31 and EMCN in femora infected with rAAV2/9-Tie-Ybx1. Scale bar 50 μm. Data are shown as the mean ± SEM. *, P < 0.05; **, P < 0.01; ***, P < 0.001 by one-way ANOVA.

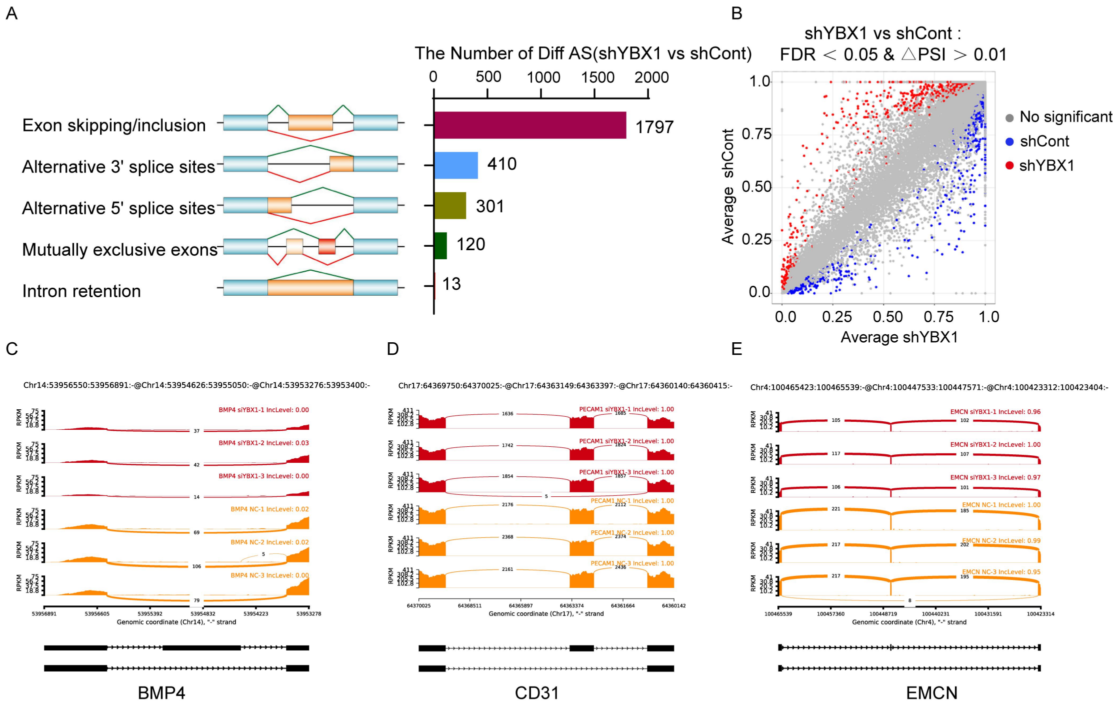

Supplemental Figure 3 Deletion of *YBX1* in endothelial cells had no effect on *BMP4*, *CD31*, and *EMCN* pre-mRNA alternative splicing. A. Schematic of pre-mRNA alternative splicing and the number of differentially spliced events in HUVECs infected with shYBX1 adenovirus or control adenovirus. B. Scatterplot of exons promoted (red circles) and repressed (blue circles) in HUVECs infected with shYBX1 adenovirus or control adenovirus. C-E. Sashimi plots of *BMP4*, *CD31* and *EMCN* in HUVECs infected with shYBX1 adenovirus or control adenovirus. n = 3 independent experiments.

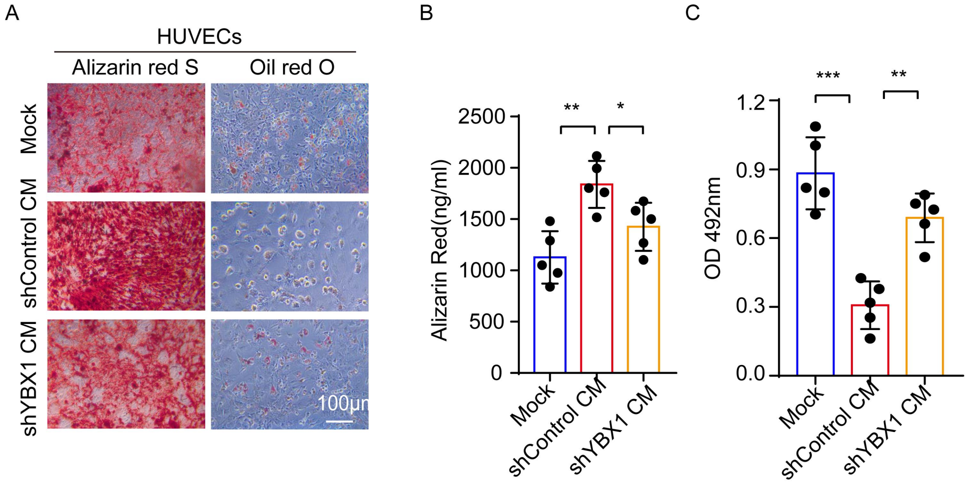

Supplemental Figure 4 HUVECs medium have an effect on osteogenic differentiation and adipogenic differentiation of BMSCs. A. Representative images of Alizarin Red staining (left panel) and Oil Red O staining (right panel) in BMSCs treated with conditioned medium from HUVECs infected with shYBX1 adenovirus or control adenovirus. B-C. Quantification of calcium mineralization based on Alizarin Red staining (B) and quantification of Oil Red O based on Oil Red O staining (C) in BMSCs treated with conditioned medium. n = 5 independent experiments. Scale bar 100 μm. Data are shown as the mean ± SEM. *, $P < 0.05$; **, $P < 0.01$; ***, $P < 0.001$ by one-way ANOVA.

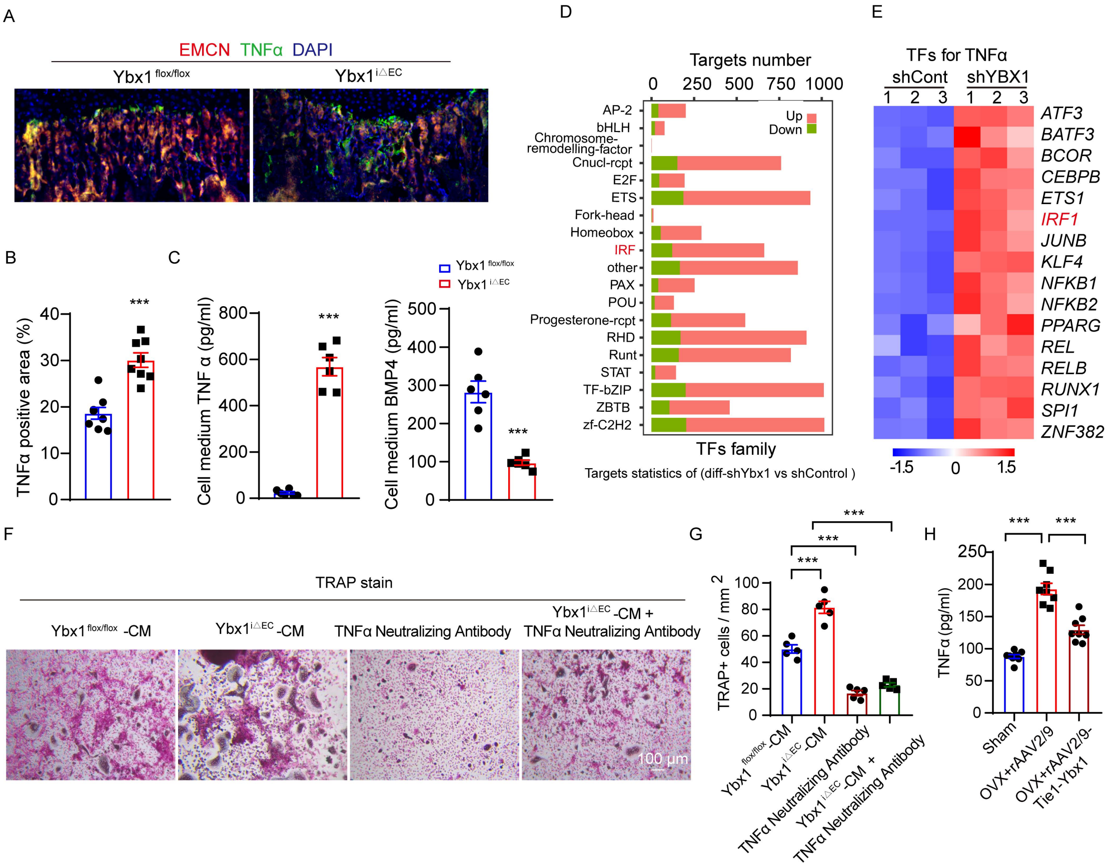

Supplemental Figure 5 Deletion of *Ybx1* in endothelial cells activated TNFα expression. A-B. Representative images and quantitation of EMCN (red) and TNFα (green) immunostaining in femora from endothelial-specific *Ybx1* knockout mice (*Ybx1*$^{i\triangle EC}$) and their littermate controls (*Ybx1*$^{flox/flox}$). C. ELISA analysis of TNFα (left) and BMP4 (right) levels in cell medium from isolated *Ybx1*$^{i\triangle EC}$ and *Ybx1*$^{flox/flox}$ endothelial cells. D. The altered transcription factors family and the number of target genes of transcription factors in endothelial cells infected with shYBX1 adenovirus or control adenovirus. E. Heat-map of altered transcription factors involves regulating TNFα between endothelial cells infected with shYBX1 adenovirus or control adenovirus. F-G. Representative images of TRAP staining (F) and quantification (G) of staining of osteoclasts treating with conditioned medium of primary endothelial cells from *Ybx1*$^{i\triangle EC}$ and *Ybx1*$^{flox/flox}$ mice, with or without TNFα neutralizing antibody in it. H. ELISA analysis of TNFα levels in serum from sham, OVX, and OVX mice injected with AAV2/9. sup-*Tie1*-*Ybx1*. In B and H, n = 8 independent experiments. In C and G, n = 5 independent experiments. Scale bar 100 µm. Data are shown as the mean ± SEM. ***, P < 0.001 by Student's t test (B and C) and two-way ANOVA.

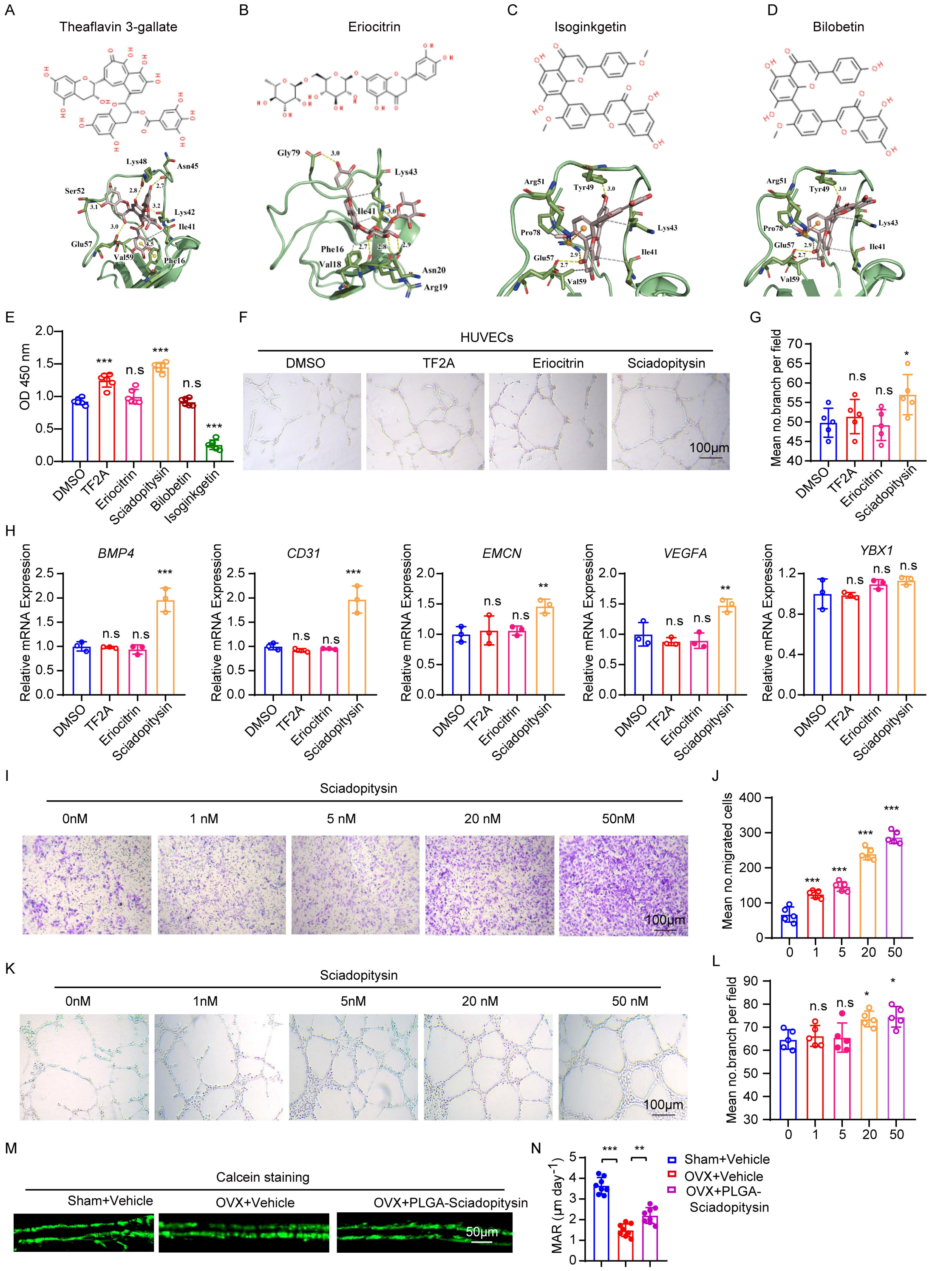

Supplemental Figure 6 Screening for natural small molecular compounds that activate *YBX1* expression. A-D. The Theaflavin-3-gallate (TF2A), Eriocitrin, Isoginkgetin, and Bilobetin structure (upper panel) and the key residues for interaction between them and YBX1 (bottom panel). E. CCK8 assay analysis of the HUVECs viability. F-G. Representative images (F) and relative quantification (G) of tube branch numbers of HUVECs treated with TF2A, Eriocitrin and Sciadopitysin. H. *BMP4*, *CD31*, *EMCN*, *VEGFA* and *YBX1* mRNA expression in HUVECs treated with TF2A, Eriocitrin and Sciadopitysin. I-L. Representative images and relative quantification of migrating cells (I and J), tube branch numbers (K and L) of HUVECs treated with different concentrations of sciadopitysin. M-N. Representative images (M) and quantification (N) of calcein double labeling in femora of sham, OVX, and OVX injected with PEG-PLGA nanoparticles carrying sciadopitysin (CD31 modified), n=8 group. In E, G, J and L, n = 5 independent experiments. In H, n = 3 independent experiments. Scale bar 100 μm and 50 μm. Data are shown as the mean ± SEM. *, P < 0.05; **, P < 0.01; ***, P < 0.001 by one-way ANOVA.

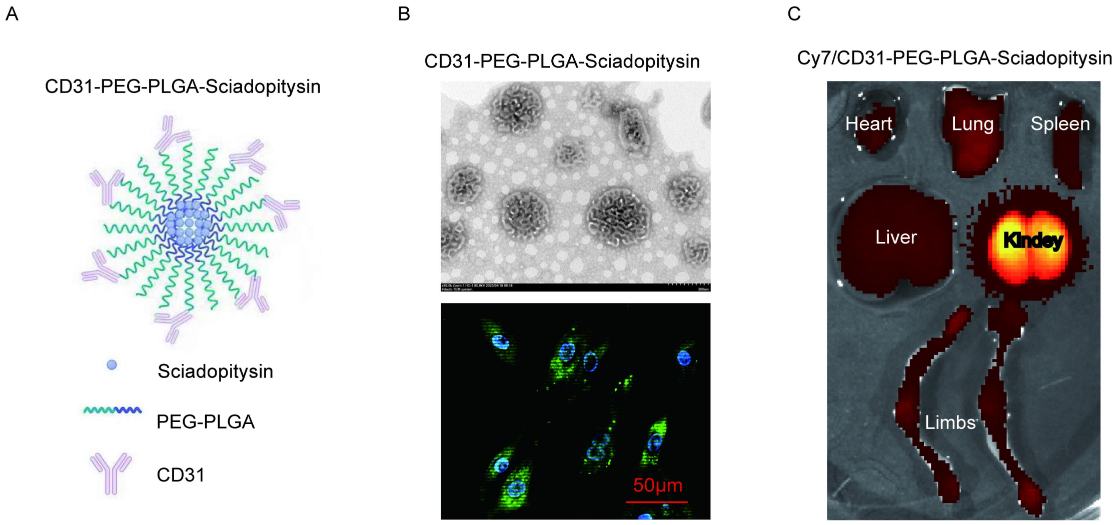

Supplemental Figure 7 PEG-PLGA-sciadopitysin nanoparticles effectively target endothelial cells. A. Schematic of CD31-modified PEG-PLGA-sciadopitysin nanoparticles. B. Representative image of scanning electron microscopy of CD31-modified PEG-PLGA-sciadopitysin nanoparticles (up panel) and the nanoparticles were transferred into HUVECs (bottom panel). C. Representative ex vivo fluorescence images of heart, lung, spleen, liver, kidneys, and hind limbs of 8-week-old mice, which were injected with Cy7/CD31-modified PEG-PLGA-sciadopitysin nanoparticles by tail vein and sacrificed 24 h post injection. Scale bar 50 μm.

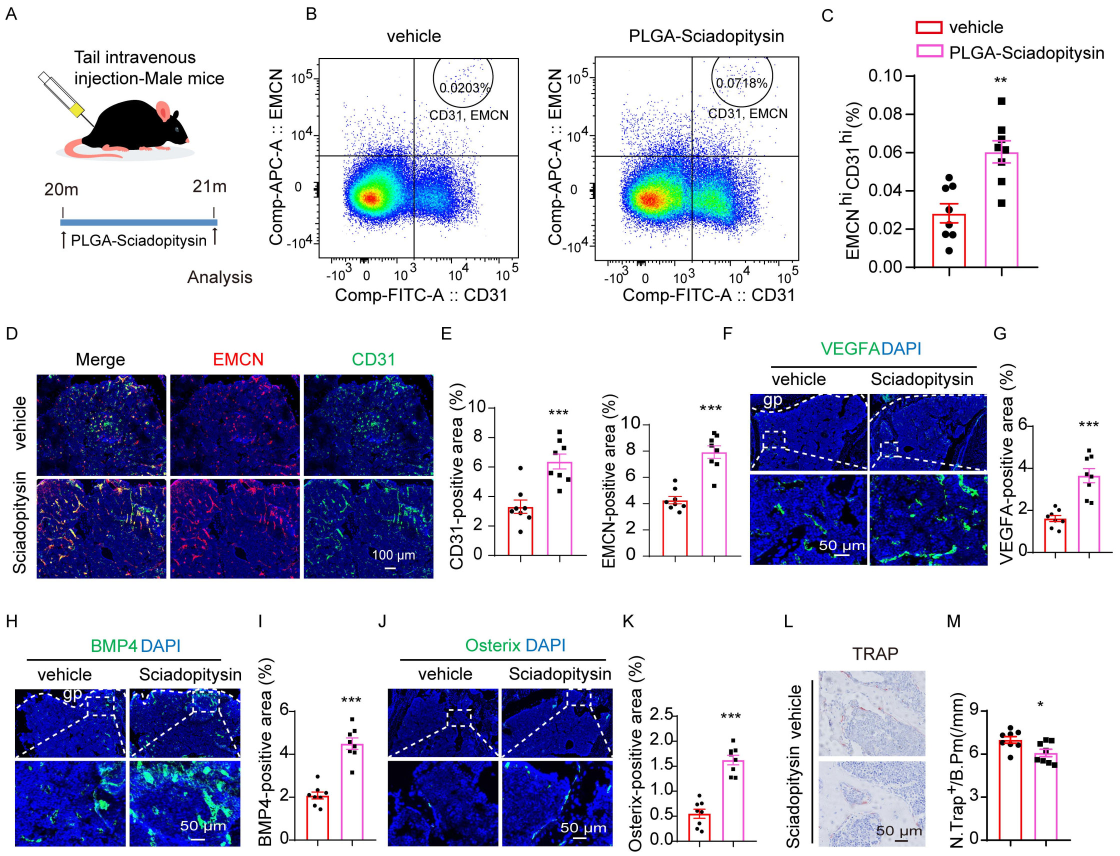

Supplemental Figure 8 PEG-PLGA nanoparticles carrying sciadopitysin enhance angiogenesis-dependent bone formation in aged male mice. A. Schematic diagram of treating aged male mice with sciadopitysin. B-C. FACS analysis dot plot (B) and quantification (C) of $CD31^{hi}EMCN^{hi}$ ECs from aged male mice injected with vehicle (PEG-PLGA nanoparticles) and PEG-PLGA nanoparticles carrying sciadopitysin (CD31 modified). D-E. Representative images (D) and quantitation (E) of CD31 (green) and EMCN (red) immunostaining in each group. Scale bar, 100 μm. F-G. Representative images (F) and quantitation (G) of VEGFA (green) immunostaining in each group. Scale bar, 50 μm. H-I. Representative images (H) and quantitation (I) of BMP4 (green) immunostaining in each group. Scale bar, 50 μm. J-K. Representative images (J) and quantitation (K) of $Osterix^{+}$ (green) immunostaining in each group. Scale bar, 50 μm. L-M. Representative images (L) of TRAP staining and quantification (M) of $TRAP^{+}$ cells in trabecular bone surfaces. Scale bar, 50 μm. n = 8 mice in each group. n = 2 independent experiments. Data are shown as the mean ± SEM. *, $P < 0.05$; **, $P < 0.01$; ***, $P < 0.001$ by student's t test.
