# Supplementary figures and images for: YBX1 promotes type H vessel–dependent bone formation in an m5C-dependent manner
Source: JCI Insight. 2024 Feb 22;9(4):e172345. doi: 10.1172/jci.insight.172345 (PMC11143935; doi:10.1172/jci.insight.172345)

Figure 4. G

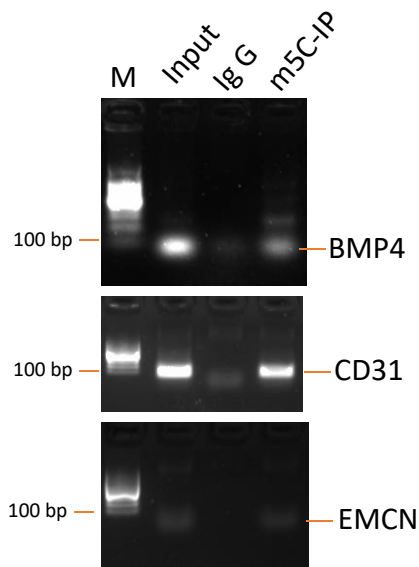

Figure 4. H

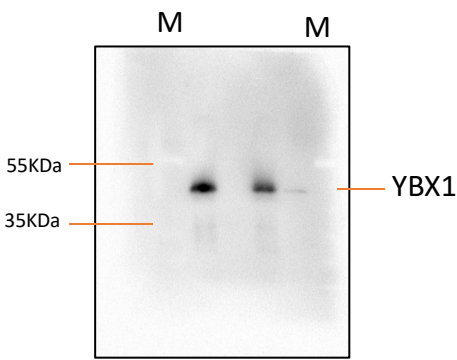

Figure 4. I

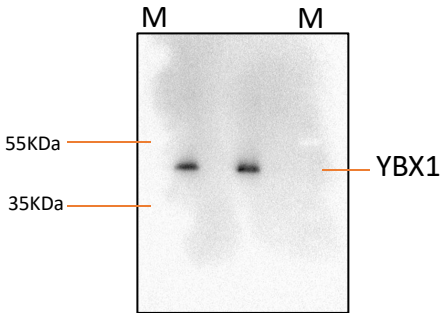

Figure 4. N

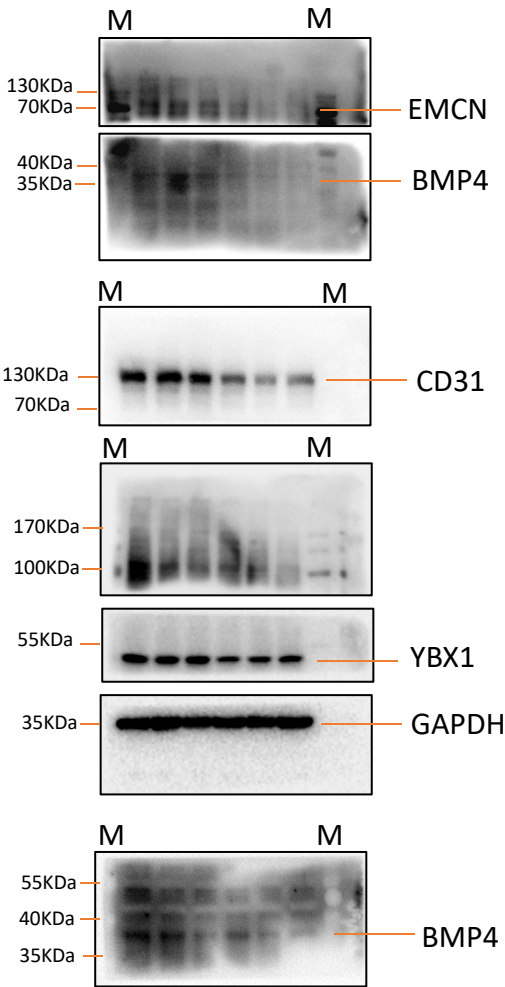

Figure 6. C

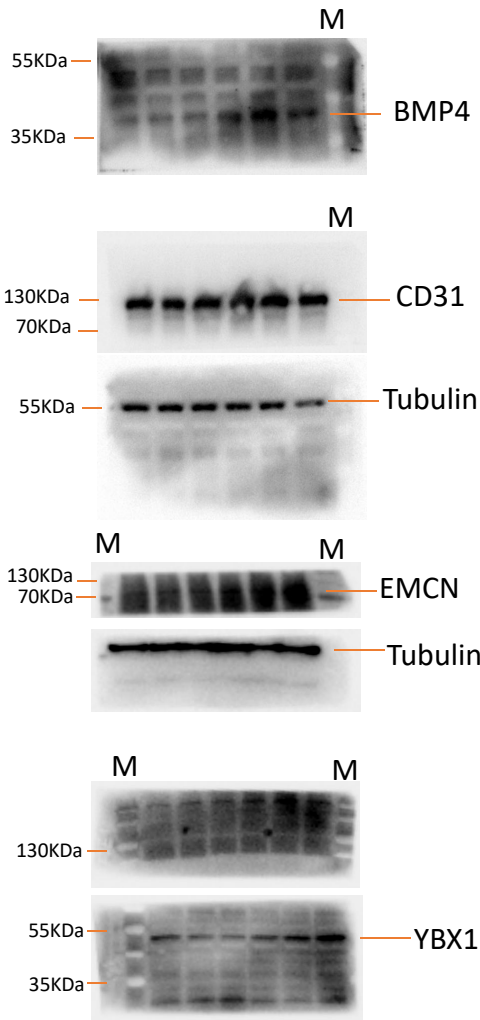

Figure 7. D

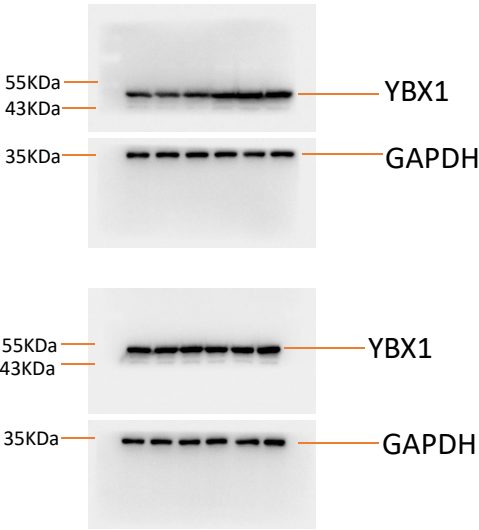

Supplement: Unedited blot and gel images [file jciinsight-9-172345-s058.pdf]
